# Characteristic Volatile Fingerprints and Odor Activity Values in Different Citrus-Tea by HS-GC-IMS and HS-SPME-GC-MS

**DOI:** 10.3390/molecules25246027

**Published:** 2020-12-19

**Authors:** Heting Qi, Shenghua Ding, Zhaoping Pan, Xiang Li, Fuhua Fu

**Affiliations:** 1 Longping Branch Graduate School, Hunan University, Changsha 410125, China; qiheting@hnu.edu.cn (H.Q.); shhding@hotmail.com (S.D.); pzpjl@163.com (Z.P.); lixiang666@hnu.edu.cn (X.L.); 2 Provincial Key Laboratory for Fruits and Vegetables Storage Processing and Quality Safety, Agricultural Product Processing Institute, Hunan Academy of Agricultural Sciences, Changsha 410125, China; 3 Hunan Province International Joint Lab on Fruits & Vegetables Processing, Quality and Safety, Changsha 410125, China

**Keywords:** citrus-tea, volatile components, HS-GC-IMS, HS-SPME-GC-MS, orthogonal partial least squares discrimination analysis, OAV

## Abstract

Citrus tea is an emerging tea drink produced from tea and the pericarp of citrus, which consumers have increasingly favored due to its potential health effects and unique flavor. This study aimed to simultaneously combine the characteristic volatile fingerprints with the odor activity values (OAVs) of different citrus teas for the first time by headspace gas chromatography-ion mobility spectrometry (HS-GC-IMS) and headspace solid-phase microextraction-gas chromatography-mass spectrometry (HS-SPME-GC-MS). Results showed that the establishment of a citrus tea flavor fingerprint based on HS-GC-IMS data can provide an effective means for the rapid identification and traceability of different citrus varieties. Moreover, 68 volatile compounds (OAV > 1) were identified by HS-SPME-GC-MS, which reflected the contribution of aroma compounds to the characteristic flavor of samples. Amongst them, the contribution of linalool with sweet flower fragrance was the highest. Odorants such as decanal, *β*-lonone, *β*-ionone, *β*-myrcene and *D*-limonene also contributed significantly to all samples. According to principal component analysis, the samples from different citrus teas were significantly separated. Visualization analysis based on Pearson correlation coefficients suggested that the correlation between key compounds was clarified. A comprehensive evaluation of the aroma of citrus tea will guide citrus tea flavor quality control and mass production.

## 1. Introduction

Citrus tea is a unique tea product made by adding fermented tea to citrus shells and then redrying them together. It has the characteristics and functional properties of citrus peel and tea, providing a mellow taste and citrus aroma, and has become rapidly popular in the Chinese tea market. Citrus tea contains several functional components, such as *D*-limonene, hesperidin, tea polyphenols and tea polysaccharide, which have been proven to possess multiple health-promoting effects, including antioxidation [1], anti-aging [2] and hypolipidemic efficacies [3].

In addition to its functional ingredients, the flavor of citrus tea is also crucial to consumers’ choice. Citrus tea has the flavors of both citrus and tea, and the volatile organic compounds (VOCs) with aroma components mainly include olefins, esters and alcohols. The flavor of citrus tea is influenced by many intrinsic and extrinsic factors, such as variation of cultivar resources, processing technologies and storage conditions. In terms of raw materials, Pu’er tea and *C. reticulata* “Chachi” peel are commonly used to produce citrus tea, which has a large market scale. Jiang et al. [4] found that the VOCs of Ganpu tea prepared by citrus of different maturity were significantly different. When processing citrus tea, the operating parameters are optimized to improve the quality of citrus tea. Zheng et al. [5] showed that ultrasonic treatment could accelerate the aging process and improve the flavor of Ganpu tea. In terms of storage, different storage conditions and time will result in some changes in the flavor of the same citrus tea. At present, the raw material that is mainly used in producing citrus tea is Chachi peel, which is widely planted in Guangdong Province in China. Meanwhile, Hunan Province is rich in citrus varieties, and the development of citrus tea can make use of surplus citrus resources. Flavor selection and adjustments of different citrus tea varieties are conducted to meet the tastes and preferences of different consumers. Therefore, the development of new citrus tea products by preparing citrus tea from different citrus resources and comparing their similarities and differences in flavor is of great significance.

Headspace gas chromatography-ion mobility spectrometry (HS-GC-IMS) is a new technology based on gas chromatography and ion mobility spectrometers, which can detect the fingerprint of VOCs in unpretreated liquid or solid samples [6], thus preserving the flavor of the samples more completely. The basic principle of IMS technology is to separate ions according to the different migration rates when they pass through the gas in the electric field at ambient pressure [7] (pp. 177–197). In recent years, HS-GC-IMS has been extensively used to analyze volatile compounds in the food field, such as detecting the authenticity of traditional Chinese medicine [8], adulterated honey [9], properties of raspberry wines [10] and *Tricholoma matsutake* Singer [11]. IMS technology has the advantages of no pretreatment and high detection sensitivity, and the fingerprint generated by the system can be used to observe the differences between samples more intuitively and conveniently. However, HS-GC-IMS instruments cannot accurately determine the volatile concentration. Thus, combining IMS with other devices is a suitable way to increase its advantages and produce an excellent evaluation result. To the best of our knowledge, IMS technology cannot be used to obtain information about the key odorant active compounds.

Odor activity value (OAV) calculation is widely used in the screening and identification of key odorant active compounds in food, such as major odorants in cempedak [12], green tea beverages [13] and prawns [14]. The common determination method of VOCs is headspace solid-phase microextraction–gas chromatography–mass spectrometry (HS-SPME-GC-MS), which can perform comparative identification according to the retention index (RI) and accurate quantitative analysis by adding internal or external standards. Accordingly, OAV can be calculated based on the HS-SPME-GC-MS data to further screen key aroma compounds. At present, investigations focusing on how key compounds contribute to citrus tea aroma are quite limited.

Thus, in this study, the peel of 19 types of unripe citrus fruits mainly cultivated in Hunan Province and dark tea from Anhua County (which is known as the hometown of tea) were used as raw materials to prepare citrus tea. The aroma profiles of citrus tea were explored by using HS-GC-IMS and HS-SPME-GC-MS. The results of fingerprint and similarity analysis were compared directly and quickly to facilitate the comparison of the aroma in different citrus teas. Furthermore, VOCs were quantitatively analyzed, and the OAVs were calculated. The key aroma compounds were identified, and the results could be used to regulate the aroma of citrus tea in mass production.

## 2. Results and Discussion

### 2.1. Identification of Volatile Substances in Citrus-Tea

The volatile components of 19 types of citrus tea were obtained by HS-GC-IMS combined with HS-SPME-GC-MS analysis, and a total of 382 components were identified. A total of 59 peaks (Figure S1) and 44 identified components by IMS library listed in Table S1, which included 7 terpenes, 9 alcohols, 11 aldehydes, 4 esters, 7 ketones, 2 acids, 2 furans, dimethyl disulfide and 2-ethyl-6-methylpyrazine. Moreover, 358 types of VOCs were analyzed by HS-SPME-GC-MS (Table S2). These compounds could be divided into seven categories: 16 phenolic acids, 28 esters, 69 alcohols, 26 aldehydes, 34 ketones, 131 hydrocarbons, 3 ethers, 25 benzene derivatives and 26 nitrogen compounds. Therefore, GC-MS identified more compounds than HS-GC-IMS.

**Figure 1:**
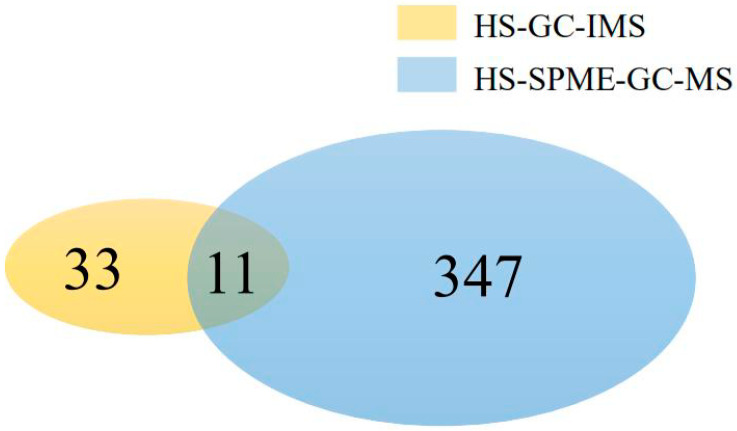
Venn diagram of volatile compounds detected via headspace gas chromatography-ion mobility spectrometry (HS-GC-IMS) and headspace solid-phase microextraction-gas chromatography-mass spectrometry (HS-SPME-GC-MS).

The two techniques identified 11 overlapping compounds (Figure 1), including limonene, linalool, octanal, decanal, pinene, *β*-myrcene, *γ*-terpinene, *β*-ocimene, etc. In previous studies [4], these VOCs were recognized as the main flavor compounds in citrus tea. Some small molecules of C_2_~C_7_ compounds, including acetone, 2-butanone, acetoin, pentanal, *γ*-decalactone, 2-acetylfuran and so on, could not be detected by HS-SPME-GC-MS, but they were detected by HS-GC-IMS. These differences were due to the characteristics of GC-IMS. Although both instruments are in series with the gas chromatography, the foci of the two instruments are different. HS-SPME-GC-MS is more focused on qualitative and quantitative accuracy, whereas HS-GC-IMS is more focused on sample discrimination. HS-GC-IMS does not respond to alkanes, and VOCs with high molecular weight, such as benzene derivatives and nitrogenous compounds, are difficult to detect. The working conditions of atmospheric pressure and constant temperature and the method of collecting the headspace gas facilitate the detection of small molecules. However, the limitations of the currently available library for HS-GC-IMS hinder the qualitative analysis of the VOCs of citrus tea. Therefore, the signals of some substances that are not included in the database were detected and were not shown in the table. Some single compounds, including α-pinece, carvone and *trans*-caryophyllene, showed multiple signals in the drift time scale, which can be explained by the formation of adducts between the analyzed ions and neutral molecules such as dimers and hydrates during the movement through the IMS drift tube [15]. The formation of such clusters is particularly common in compounds with high strong proton affinity [16]. In this study, most of the aldehydes and ketones, as well as *β*-ocimene, limonene, *β*-pinene and ethyl acetate compounds, have two peaks due to the presence of these two monomers.

**Figure 2:**
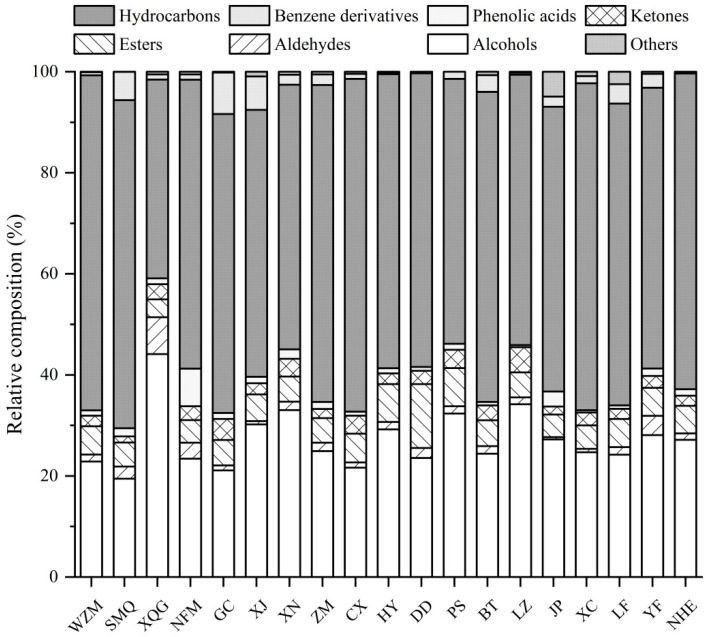
Relative composition (%) of classes of volatile components in citrus tea based on HS-SPME-GC-MS data. WZM: citrus-tea made from Wenzhou Tangerine Dafen No. 4 and Tianjian tea; SMQ: citrus-tea made from Shimeng orange and Tianjian tea; XQG: citrus-tea made from Xiaoqing orange and Tianjian tea; NFM: citrus-tea made from Nanfeng orange and Tianjian tea; GC: citrus-tea made from Gongchuan orange and Tianjian tea; XJ: citrus-tea made from Xinjing orange and Tianjian tea; XN: citrus-tea made from Xinnv Ponkan and Tianjian tea; ZM: citrus-tea made from Zaomi Ponkan and Tianjian tea; CX: citrus-tea made from Zaomi Ponkan and Tianjian tea; HY: citrus-tea made from Huyou and Tianjian tea; DD: citrus-tea made from Daidai lime and Tianjian tea; PS: citrus-tea made from Pushi Cheng and Tianjian tea; BT: citrus-tea made from Bingtang orange and Tianjian tea; LZ: citrus-tea made from Luzai honey orange and Tianjian tea; JP: citrus-tea made from Jinpenyou and Tianjian tea; XC: citrus-tea made from Tarocco blood orange and Tianjian tea; LF: citrus-tea made from Langfeng navel orange and Tianjian tea; YF:citrus-tea made from Yuanfeng navel orange and Tianjian tea; NHE: citrus-tea made from Newhall navel orange and Tianjian tea.

The aroma of citrus tea was formed by a complex combination of VOCs (Figure 2). Hydrocarbons accounted for a high proportion in most citrus tea, followed by alcohols, esters and ketones. Specifically, citrus-tea made from Xiaoqing orange and Tianjian tea (XQG) contained the highest proportion of alcohols (44.11%) and aldehydes (7.30%) but had the lowest proportion (39.35%) of hydrocarbons. The highest proportion of hydrocarbons in citrus-tea made from Wenzhou Tangerine Dafen No. 4 and Tianjian tea (WZM) was 66.34%. The proportion of esters in citrus-tea made from Daidai lime and Tianjian tea (DD) was 12.65%, which was higher than those in other samples. Amongst different pericarps of citrus varieties, the concentration of VOCs is significantly different [17]. Hence, the different proportion of VOCs in citrus tea is attributed to the particularity of diverse citrus species.

### 2.2. Citrus-Tea Discrimination by Characteristic Volatile Fingerprints

#### 2.2.1. Differences in the Characteristic Volatile Fingerprints of Citrus-Tea

**Figure 3:**
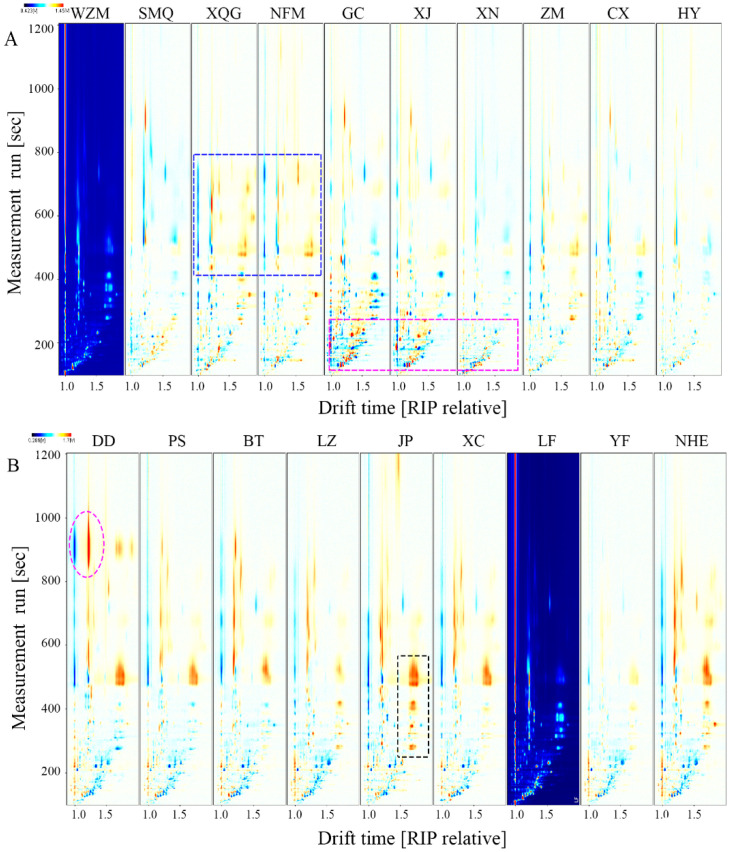
Two-dimensional topographic images of citrus tea: WZM, SMQ, XQG, NFM, GC, XJ, XN, ZM, CX and HY are shown in (**A**); DD, PS, BT, LZ, JP, XC, LF, YF and NHE are shown in (**B**).

The FlavourSpec^®^ instrument was used to generate data in the form of a 2D spectrum (Figure 3) for comparison. The red vertical line at abscissa 1.0 is the peak of reaction ion, and each point on the right represents different VOCs. The difference comparison model was used to compare the differences in citrus tea samples of the same genus. Considering a large number of samples, 19 citrus tea samples were divided into two groups. Group A consists of *C. reticulata*, *C. poonensis*, *C. unshiu* and *C. maxima*: WZM, citrus-tea made from Shimeng orange and Tianjian tea (SMQ), XQG, citrus-tea made from Nanfeng orange and Tianjian tea (NFM), citrus-tea made from Gongchuan orange and Tianjian tea (GC), citrus-tea made from Xinjing orange and Tianjian tea (XJ), citrus-tea made from Xinnv Ponkan and Tianjian tea (XN), citrus-tea made from Zaomi Ponkan and Tianjian tea (ZM), citrus-tea made from Zaomi Ponkan and Tianjian tea (CX) and citrus-tea made from Huyou and Tianjian tea (HY).Whilst group B consists of *C. sinensis*, *C. aurantium* and *C. junos*: DD, citrus-tea made from Pushi Cheng and Tianjian tea (PS), citrus-tea made from Bingtang orange and Tianjian tea (BT), citrus-tea made from Luzai honey orange and Tianjian tea(LZ), citrus-tea made from Jinpenyou and Tianjian tea (JP), citrus-tea made from Tarocco blood orange and Tianjian tea (XC), citrus-tea made from Langfeng navel orange and Tianjian tea (LF), citrus-tea made from Yuanfeng navel orange and Tianjian tea (YF) and citrus-tea made from Newhall navel orange and Tianjian tea (NHE). WZM and LF were selected as reference samples for the two groups of spectrograms, and the color of the compounds with the same content after deduction was white. The red spots in the sample indicated that the content of the compounds was higher than that of the reference sample, and the darker the red, the higher the content. By contrast, blue dots indicated that the concentration of the compounds was lower than that of reference samples, and the darker the blue color, the lower the sample content.

Most of the VOCs were located in the region where the retention time was 0–1000 s and the drift time was 1.0–1.5 s. The VOCs of XQG and NFM in 400–800 s were relatively higher than those of other samples. GC, XJ and XN were significantly different in flavor substances within 0–400 s compared with others in group A. Moreover, YF and LF had a similarity of 83%. GC and XJ were 78% similar according to the GC-IMS built-in analysis software (Figure S2). Compared with other citrus tea in group B, JP showed more red spots in the black dotted box (Figure 3B). In the retention time range of 800–1000 s, the content of VOCs in DD was higher than that of others. The peak locations and quantities of citrus tea were approximately the same in the spectra. By contrast, the peak strength showed significant differences, indicating that the contents of each VOC varied with the germplasms of citrus peels.

Considering that the 2D spectrum shows the overall view of the VOCs, it is difficult to analyze the overlapping substances in the image accurately. Therefore, the fingerprints (Figure S3) of citrus tea were formed on the basis of all signal peaks. In the fingerprints, each row represents all volatile peaks of a sample, and each column represents the signal peak of the same substance in different samples. The spots range from blue to red, indicating low to high contents of volatile substances. The consensus identifies 59 signal peaks and marks them with their existing names, whilst numbers represent unknown substances. The fingerprint of each citrus tea was divided into four parts for observation.

Area A was mainly composed of terpenes, including limonene, *β*-ocimene, myrcene and pinene. Amongst them, monoterpenoids are synthesized [18] from acetyl-coenzyme and pyruvate, such as linalool (citrus-like), linalool oxide (flower) and geraniol (rose-like). These precursors are mainly provided by carbohydrate pools present in plastids and cytoplasm [19]. Limonene, *β*-ocimene-M, *β*-pinene and acetone in area A were abundant in all samples. In particular, linalool, *β*-pinene-M and α-pinene were relatively low in DD. The citrus for DD preparation is a variety of sour orange (*C. aurantium* L.), which is mainly cultivated in Japan and Southern China. Aliphatic and olefin non-terpenoid compounds such as esters and aldehydes are mainly found in *C. aurantium* [20]. The relatively lower contents of linalool and α-pinene observed in DD than other samples are consistent with previous reports on citrus oil of DaiDai [21].

Area B was mainly composed of aldehydes, ketones and alcohols. Aldehyde is a kind of secondary metabolite formed during orange ripening, which provides a fruit aroma similar to citrus [22]. Because their odor threshold is low, it can also affect the overall aroma of the sample at low content. As can be seen in the red-framed area of Figure S3B, benzaldehyde, 1-hexanol, 2-hexanone and E-2-hexenol have higher contents in SMQ. Pyrazines are mainly found in cheese and have a nut-like aroma [23]. Moreover, 2-ethyl-6-methylpyrazine is a typical Maillard reaction compound [24], so it could be formed during the process of de-enzyming. Low-carbon saturated ketones such as cyclohexanone-D and 2,3-butanedione are the main volatile components of cheese [25] and have a creamy aroma. Methyl N-methyl-anthranilate has a warm fruit aroma in citrus and has been reported as an aromatic biomarker of Ganpu tea because it is a characteristic aroma substance of *C. reticulata* “Chachi” [26], which is the raw material of Ganpu tea. In this study, methyl N-methyl-anthranilate has a high content in NFM but a very low content in other citrus tea, suggesting that *C. reticulata Blanco* “Kinokuni” and *C. reticulata* “Chachi” have the same flavor markers. Furthermore, the contents of α-terpentinol, *β*-citronellol and ethyl acetate were higher in XQG.

In area C, VOCs such as dimethyl disulfide, phenyl ethyl acetate, furfurol-D, 2-pentanone, 2-butanone and 2-furfuryl mercaptan were concentrated in GC and XJ with dark red color in the green-framed area of Figure S3C, indicating extremely high content. Their contents were obviously different in other varieties of citrus tea. Volatile sulfur compounds are vital aroma-inducing compounds with low threshold and strong odor and are widely distributed in food products [27]. Dimethyl sulfide [28] has an ‘asparagus”, “corn” and “molasses” aroma and is considered a beneficial compound at low contents. Moreover, 3-Methyl-butyraldehyde-M and 2-ethylfuran have low content in all samples. The formation of these malty smelling aldehydes during the processing of tea leaves might be explained by a reaction of their parent amino acids (valine, isoleucine and leucine) with α-dicarbonyl compounds, such as oxidized tea polyphenols (ortho-quinones) [29] or methylglyoxal [30], as postulated earlier.

Most VOCs in area D, such as octanal, 1-hexanol, 1-pentanor, acetoin-M, 2-methylbutanar and ethanol, have a low content in GC and XJ. During the drying of citrus tea, furans were formed by dehydration of deoxidized aldose and ketose, such as the thermal degradation of fructose and glucose [31]. The content of 2-acetylfuran in WZM, XQG and SMQ was relatively low. Previous studies showed that prominent increases in *cis*-geraniol, *trans*-*β*-farnesene and *β*-caryophyllene were found in DaiDai essential oil during storage [32]. This could be the reason for the highest geraniol content in DD. *γ*-Decalactone, with a greasy peach aroma that resembles sweet milk, had the highest content in JP. Amongst the key aroma components of fruit, the biosynthesis of lactone is related to the *β*-oxidation pathways, such as *γ*-decalactone in peach, *δ*-octalactone in pineapple and *γ*-octalactone in coconut [33].

#### 2.2.2. Establishment of Models for Citrus-Tea

**Figure 4:**
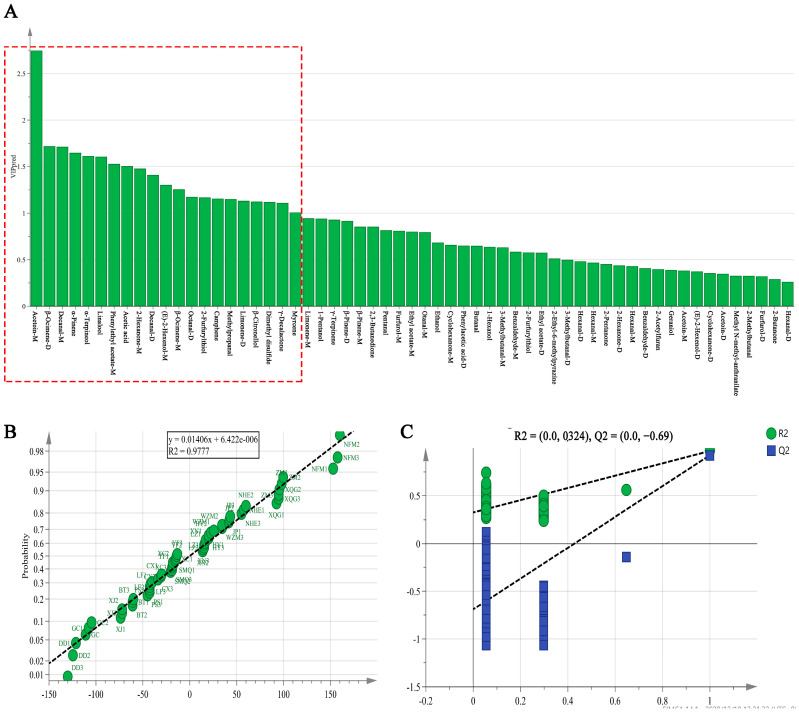
Orthogonal partial least squares discrimination analysis (OPLS-DA) of 19 types of citrus-tea. (**A**) The variable importance for the projection (VIP) predictive of the volatile organic compounds (VOCs); (**B**) Normal probability of mode; (**C**) Permutations plot (*n* = 200) of VOCs in citrus-tea.

Supervised orthogonal partial least squares discrimination analysis (OPLS-DA) contributes to the visualization of high-dimensional data and discriminant analysis of potential metabolites associated with metabolic changes. In addition, the variable importance for the projection (VIP) predictive and substitution tests can be used to evaluate the performance of the model in OPLS-DA [34]. VIP is generally used to evaluate the contributions of X-variables to a model; variables with VIP > 1 are generally considered to be important variables. The greater the VIP value, the more significant the difference in the volatile compound in different citrus teas. The VIP values of 21 compounds were > 1 (red box in Figure 4A), including almost all terpenes, aldehydes, alcohols and furans. Consistent with previous observations, these VOCs are markers by which to distinguish different samples of citrus tea. In Figure 4B, the R^2^ of model was 0.978, which meant that the fit of the model was good.

To validate the robustness of the model, a permutation test (Figure 4C) was performed. A permutation test (*n* = 200) is a method of performing the random arrangement of sample data and then statistical inference, which can increase the number of samples in the model [35]. Generally, to evaluate the performance of a regression model, the prediction ability parameter (Q^2^) and the goodness of fit value (R^2^) are used. The test showed that all of the Q^2^ and R^2^ values in the permutation test were lower than the Q^2^ and R^2^ in raw values. Furthermore, the regression line slopes of R^2^ and Q^2^ were >1 and the regression line intercept of Q^2^ was negative, which demonstrated both high goodness of fit and high prediction ability [36]. Therefore, the HS-GC-IMS model was confirmed as being suitable for identifying varieties of citrus tea.

#### 2.2.3. Rapid Identification of Citrus-Tea by HS-GC-IMS

In general, the composition and content of characteristic flavor substances of different citrus teas also present corresponding differences due to the influence of citrus by breed, growth environment, weather, harvesting and other factors. Lan et al. [37] conducted characteristic aroma composition measurements on *C. Junos* and found that the samples from Gaozhi County were different during the same harvest period. Therefore, the choice of different regions and varieties of citrus raw materials is particularly important. The establishment of fingerprints of citrus tea flavor in different raw materials aimed to provide a rapid and effective means of conducting quality evaluation and origin traceability control in citrus tea.

HS-GC-IMS requires less time to obtain analytical results; the three-dimensional spectrum results contain the retention time, drift time and signal intensity, which make the qualitative analysis more accurate. In addition, HS-GC-IMS data do not require complex processing; the difference between samples can be intuitively compared through the fingerprint generated by the machine, and the similarity can be determined through its built-in software. In this study, HS-GC-IMS was used for the rapid identification of the the volatiles of citrus tea, and the prediction ability and goodness of fit of the OPLS-DA model established by the HS-GC-IMS results for citrus tea were high.

### 2.3. Key Aroma Compounds in Citrus-Tea by HS-SPME-GC-MS

#### 2.3.1. Odor Activity Value of VOCs in Citrus-Tea

Although HS-GC-IMS shows the changes in the content of the compounds, their aroma contribution could not be confirmed due to precise concentration value. Consumers usually judge the acceptability of food by aroma and flavor. The odor activity of VOCs in citrus tea is one of the main sensory traits that determines the quality of the final product. The level of OAVs can reflect the contribution of VOCs to the characteristic flavor of the sample. The OAVs of aroma compounds were calculated by calculating the concentration divided by the odor thresholds [38]. Previous reports suggested that OAV > 1 indicates its contribution to the overall aroma of samples [39], and the higher the OAV, the greater the individual contribution of the compound.

As shown in Table 1, the OAVs of 79 volatile components were determined by GC-MS. Amongst them, 68 substances had an OAV > 1. In addition, OAVs of tridecanoic acid, decyl acetate, phytol and benzaldehyde were between 0.2 and 1.0. Meilgaard et al. [40] stated that the mass concentration of substances contributing to the overall aroma should be at least 20% of the threshold (OAV > 0.2). The VOCs (1.0 > OAV > 0.2) may contribute to the overall aroma due to the synergism with other flavor compounds. Decyl acetate (OAV = 0.8) collaborates with octyl acetate (OAV = 63) in contributing waxy aromas to SMQ. Similarly, phytol (OAV = 0.3–0.6) can cooperate with other aldehydes to enhance the herbal aroma.

**Figure 5:**
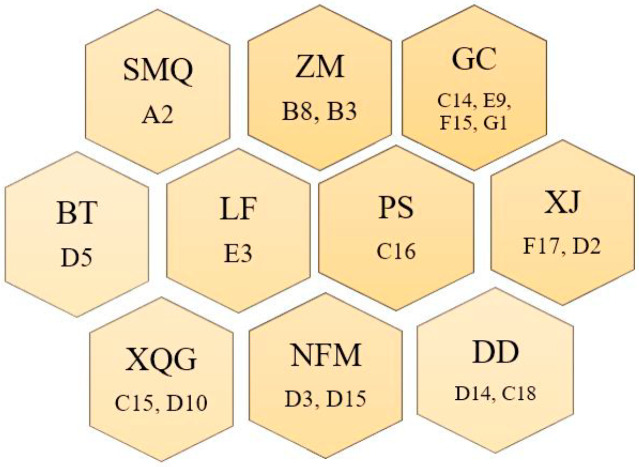
Special aromatic biomarkers (OAV > 0.2) based on HS-SPME-GC-MS data. The codes of the compounds correspond to those in Table 1.

Each type of citrus-tea showed different compounds and corresponding OAVs. In all samples, linalool, with a sweet, flowery fragrance, surpassed other odorants (OAV > 400,000). Seven compounds with an OAV > 1000 in citrus tea also contributed significantly to citrus tea. Among them, octanal, decanal and *D*-limonene have citrus aromas; *β*-lonone and *β*-ionone have violet aromas; *β*-myrcene has an elegant aroma of tea; methoxy-4-vinylphenol has a strong spicy smell. Moreover, 19 citrus tea samples have different VOCs, and 10 citrus-tea contain special aromatic biomarkers (OAV > 0.2) that play an important role in their aroma recognition (Figure 5). Based on their chemical classification, specific volatile compounds are discussed below.

Phenolic acids. Acetic acid, tridecanoic acid and n-decanoic acid were detected as acid compounds in the samples. To some extent, these acids have a high odor threshold, so they do not have a strong effect on the overall aroma of citrus tea. Thymic-smelling thymol (OAV = 1–3) and herb-smelling carvacrol (OAV = 1–27) were the phenolic substances that had a primary contribution to the aroma. Thymol has been previously identified in *C. sinensis*, *C. aurantifolia* [17], *C. junos* and *C. limon* [41]. In this study, the low OAV of thymol was also detected in CX (raw material: *C. poonensis*), GC (raw material: *C. unshiu*), LZ (raw material: *C. sinensis*) and JP (raw material: *C. junos*). Furthermore, carvacrol was found in all samples.

Esters. Esters are mainly derived from the lipoxygenase pathway and amino acid metabolism and are related to the “fruity” attribute of flavor, whose level usually increases at the late stage of ripening [42]. Seven esters with OAV > 1 were detected in citrus tea. Amongst them, wax-smelling geranyl acetate (OAV = 48–625) and rose-smelling octyl acetate (OAV = 54–308) were two major esters in most of the citrus teas. Linalyl acetate (OAV = 240) contributed significantly to the aroma of DD. Ethyl caprate (OAV = 202), found in coconut and nicotine-smelling methyl caprate (OAV = 113), was detected only in ZM and contributed to its aroma.

Alcohols. A total of 16 alcohols (OAV > 1) were detected in all samples. Linalool is the main oxygenated compound, which could be transformed to undergo enzymatic isomerization from geraniol, contributing a floral note to the characteristic aroma of all citrus teas. Similarly, spearmint-smelling (Z)-carveol (OAV = 46–535) was a major aroma volatile in all samples, whose formation results in the 6-hydroxylation of limonene by P450 enzyme [43]. Terpinen-4-ol (OAV = 14–40), 1-octanol (OAV = 35–86), p-menth-1-en-8-ol (OAV = 8–713), nerol (OAV = 20–48) and elemol (OAV = 4–43) were important contributors to the aroma of citrus tea. Compared with other samples, the OAV of *trans*-nerolidol in DD (OAV = 126) and (+)-*β*-citronellol in XQG (OAV = 1138) was higher (Table 1), and these compounds mainly have a rose-like aroma. These findings agreed with those of a previous study, which found that terpene alcohols and their oxides were the main alcohols in microbially fermented tea such as Pu’er tea [44]. These terpene alcohols and their corresponding derivatives could be produced from glycosides through hydrolysis and further oxidation during microbial fermentation treatment [45] and could provide the floral, sweet and woody notes of citrus tea.

Aldehydes. Aldehydes are secondary metabolites that are formed during the ripening of oranges and contribute to citrus-like fruity notes. Almost all aldehydes and ketones were proven to be important for aroma [46] (pp. 20–22) and increased significantly during fermentation. Fourteen aldehydes with OAV > 1 were detected in the present study, of which benzeneacetaldehyde (OAV = 647) was only detected in XJ. The oxidation of phenethyl alcohol produces phenylacetaldehyde. Phenethyl alcohol could also oxidize to phenylacetaldehyde and phenylacetic acid. Nie et al. indicated that benzaldehyde and benzyl acetate contribute to a fruity fragrance [47]. Nonanal in DD is a C_9_ aldehyde, which originates from the degradation of polyunsaturated fatty acids influenced by lipoxygenase [48]. Citrus-smelling octanal and nonanal showed the highest OAV factor of 25,880 and 92,371 in NFM, respectively. Citral (OAV = 65) was a vital contributor to the flavor of BT. Citrals are potentially derived from carotenoids, which can be formed from oxidative cleavage of the double bond between C_7_ and C_8_ of *β*-cryptoxanthin [49]. In a previous study, *L*-perillaldehyde (OAV = 76–589) originated from the degradation of cinnamic acids [22]. Similarly, the high concentration of *L*-perillaldehyde in all samples may be a similar formation mechanism. Some other aldehydes contributed to citrus aromas, including rose-smelling citronellal (OAV = 158–374,990) and (+)-citronellal (OAV = 91), floral-smelling undecanal (OAV = 31–904) and (E)-2-decenal (OAV = 129) and fatty-smelling (S)-(−)-perillaldehyde (OAV = 233–578).

Ketones. The isomers of three carvones (E1, E3, E4) and three violetones (E6, E7, E8) were identified. Amongst them, *D*(+)-carvone (OAV = 82–979) was detected in all samples except LF, whilst *L*(−)-carvone (OAV = 36) was only detected in LF. Shuang et al. [12] found that the content of the oxidation product of limonene, namely *D*-carvone, increased significantly during thermal processing via an oxidative pathway involving the unsaturated sites in limonene. (+)-Dihydrocarvone was detected in XQG, XC and ZM. *D*-Carvone and (+)-dihydrocarvone all contributed minty and herbal aromas to citrus tea. Carvones impart woody and violet notes to samples. Because the threshold of violetones [38] is low, *β*-lonone (OAV = 183,529–504,179), *β*-ionone (OAV = 347–559,318) and α-ionone (OAV = 66–223) all had a strong influence on the overall flavor of citrus tea. Other ketones, such as hawthorn-smelling 4-methylacetophenone (OAV = 686), were only detected in GC; (E, E)-3,5-octadien-2-one (OAV = 26; 29) was found in CX and JP with floral notes, and 6-methylhept-5-en-one (OAV = 1–32) was identified in five samples.

Terpenes. Terpenes are an important group that play a key role in the aroma profile of citrus tea [4]. In this study, 17 types of terpenes (OAV > 1) were identified, including 13 types of compounds with a molecular formula of C_10_H_16_, three types of terpenes with a molecular formula of C_15_H_24_ and one terpene oxide (Table 1). Most of them were monoterpenoids, and five isomers of pinene (F1, F2, F4, F5 and F6; pine-like, resinous) were detected. Amongst them, 1r-α-pinene (OAV = 390–3350) and α-pinene (OAV = 48–916) had significant contributions to the aromas of the samples. Furthermore, *β*-myrcene (OAV = 9689–29,658) (ethereal, oily) and *D*-limonene (OAV = 10875–46,910) (citrus, lemon, minty) were two important contributors to the flavor of any terpene and were present in each sample of citrus tea. *β*-Myrcene contributes a resinous note to orange, and its content and odor threshold were lower than those of *D*-limonene. Moreover, α-pinene has a positive contribution to orange peel oil [35]. *D*-Limonene is a representative compound in citrus, which usually has a high content. Previous studies have shown that *D*-limonene acts as a flavor enhancer for other flavors and is a quality marker [33]. During deep processing, *D*-limonene may be oxidized to hydrogen peroxide, which further reacts to form a large number of products, such as carvone, α-terpinol and terpinene-4-ol [25]. (Z)-*β*-Ocimene (OAV = 34–496) with green notes, *γ*-terpinene (OAV = 8–109) with citrus-like notes and *β*-caryophyllene (OAV = 44–685) with spicy notes also contributed a certain aroma to citrus tea.

Others. Other abundant phenols were determined, such as 4-ethylguaiacol (OAV = 130) and 2-methoxy-4-vinylphenol (OAV = 123–16,135). These phenols have strong spicy notes, but 4-ethylguaiacol was only detected in GC. Specifically, 1-furfurylpyrrole (OAV = 90; 181) presented filbert- and coffee-like aromas in XJ and LZ.

In addition, although OAV is a method to solve the problem of food aroma components, using OAV to express the odor contribution of volatiles only provides approximate values. Therefore, future work should perform aroma recombination experiments and omission tests to verify and provide clear evidence to prove the actual contribution of the identified effective aroma agents to the fragrance.

#### 2.3.2. Distinctive Feature Analysis Based on Principal Component Analysis

**Figure 6:**
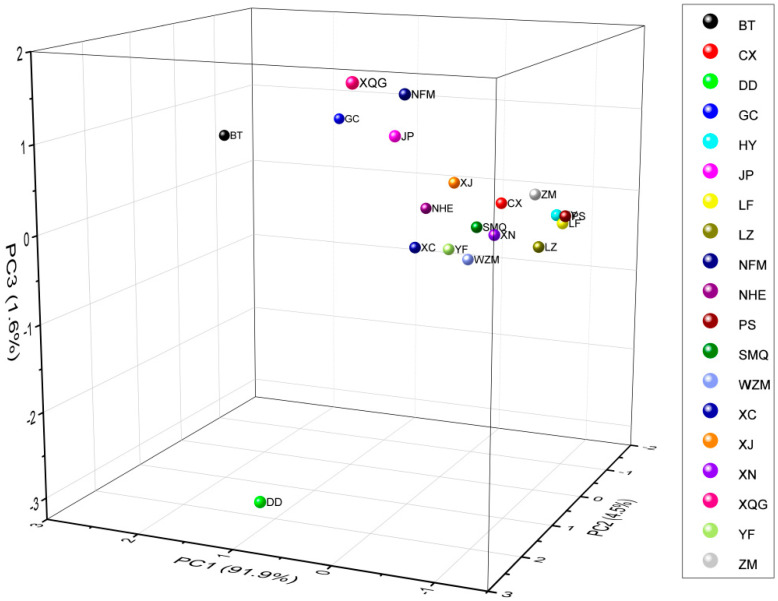
Score plot of the first three principal components of key aroma compounds (OAV > 1) based on HS-SPME-GC-MS data.

Principal component analysis (PCA) is a statistical method that transforms multiple variables into principal components by dimensionality reduction technology and is one of the most important dimensionality reduction methods [50]. The OAVs of key volatile compounds (OAV > 1) were selected for PCA to determine their contribution (Figure 6). The results showed that DD, BT, NFM, XQG, GC and JP were significantly different from other samples. In addition, LF, PS and HY were clustered, indicating that their key odorant active compounds were similar. However, DD was located outside the −3 axis of the *Z*-axis, and other samples were located within the −1–2 region of the *Z*-axis. This finding indicated that the aroma substances between DD and others had extremely different contributions.

#### 2.3.3. Correlation Coefficient Analysis of the Key Aroma Compositions

Pearson correlation coefficients (PCC) is used to measure vector similarity and the relationship of the vectors and to construct networks [51]. The closer the correlation coefficient is to 1 or −1, the stronger the correlation degree is; the closer the correlation coefficient is to 0, the weaker the correlation degree is. The hubs identified from networks are retained as key factors.

**Figure 7:**
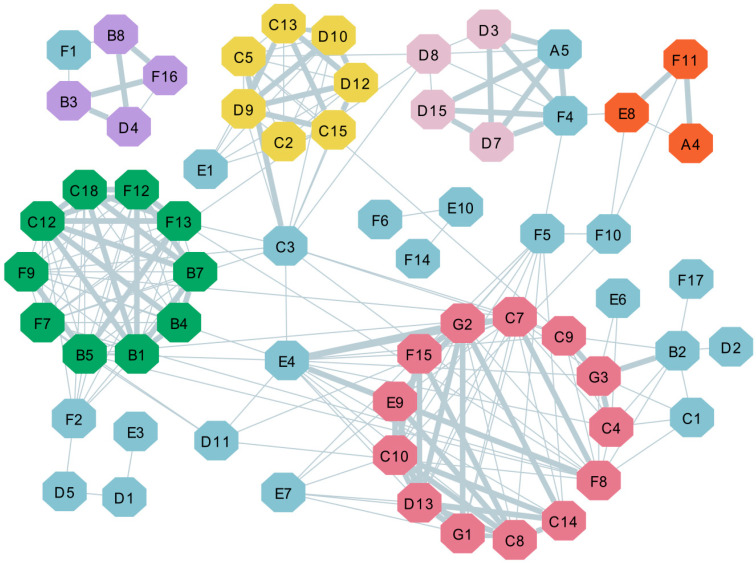
Pearson correlation visualization network for every two compounds (OAV > 1) based on GC-MS data. The nodes represent different compounds. The edge width was proportional to the r values. Grey edges represent positive correlations between two compounds. This view shows all interactions with high and medium Pearson correlation coefficients (|r| > 0.6, *p* < 0.05).

Further analysis revealed six modules in the network based on MCODE analysis (Figure 7), amongst which only the top three modules showed scores ≥4. The module of green nodes obtained the highest score (score: 8–9) as calculated via MCODE, which had a total of 10 nodes, including four esters, four olefins and two alcohols. Most of them were found in fruit and had floral aromas similar to citrus, neroli and rose. The second highest scoring cluster was the 13 modules with red nodes, in which 2-methoxy-4-vinylphenol (G2) and tridecanal (D13) had high score values. These modules had five alcohols. (Z)-carveol (C7) and (E)-carveol (C8) were isomers, and nerolidolcistrans (C4), which has a woody aroma similar to apple or rose, was found only in LZ. Moreover, 4-Ethylguaiacol (G1), 7,11-dimethyl-3-methylene-1,6,10-dodecatriene (F15) and 4-methylacetophenone (E9) were detected in GC, with 4-methylacetophenone (E9) having a characteristic hawthorn odor. Similarly, the modules composed of seven yellow nodes had high correlation. Specifically, all these VOCs existed in XQG, and (E)-2-decenal (D10) and *L*(−)-menthol (C15) were two of the characteristic VOCs of XQG. All purple compounds except citronellal (D4) only existed in ZM. Amongst them, ethyl caprate (B3) has a coconut smell, methyl caprate (B8) has a tobacco smell, 1r-α-pinene (F1) has a pine smell, and *β*-phellandrene (F16) has a pepper smell. The pink VOCs and (1S)-(−)-*β*-pinene (F4) constitute the characteristic VOCs of NFM. Nonanal (D3), decanal (D8), octanal (D7) and (+)-citronellal (D15) have a citrus aroma, whilst (1S)-(−)-*β*-pinene (F4) has a resin aroma. In summary, different modules correspond to specific citrus teas, whose VOCs constitute characteristic aroma groups, which is consistent with the above OAV analysis.

Although different statistical analyses have been used in biological and medical research areas, especially when identifying regulating genes and key microorganisms [52], few reports have used these tools to identify aroma markers and reveal the functional properties in fruit tea products. In this work, the compounds of citrus tea were visualized. The correlation between VOCs was clarified, which is of great significance for exploring the aroma composition of different citrus-tea.

## 3. Materials and Methods

### 3.1. Materials

From August to September 2019, 19 immature citrus varieties mainly planted in Hunan Province were collected. Detailed information, including the plant species, source and plucking time of citrus, is shown in Table 2. Dark tea was provided by Whiteshaxi Tea Company, Ltd. (Yiyang, China), and the detailed processing procedure, including de-enzyming, rolling, pile fermentation and drying, was described by Cao et al. [53].

### 3.2. Citrus-Tea Preparation

**Figure 8:**
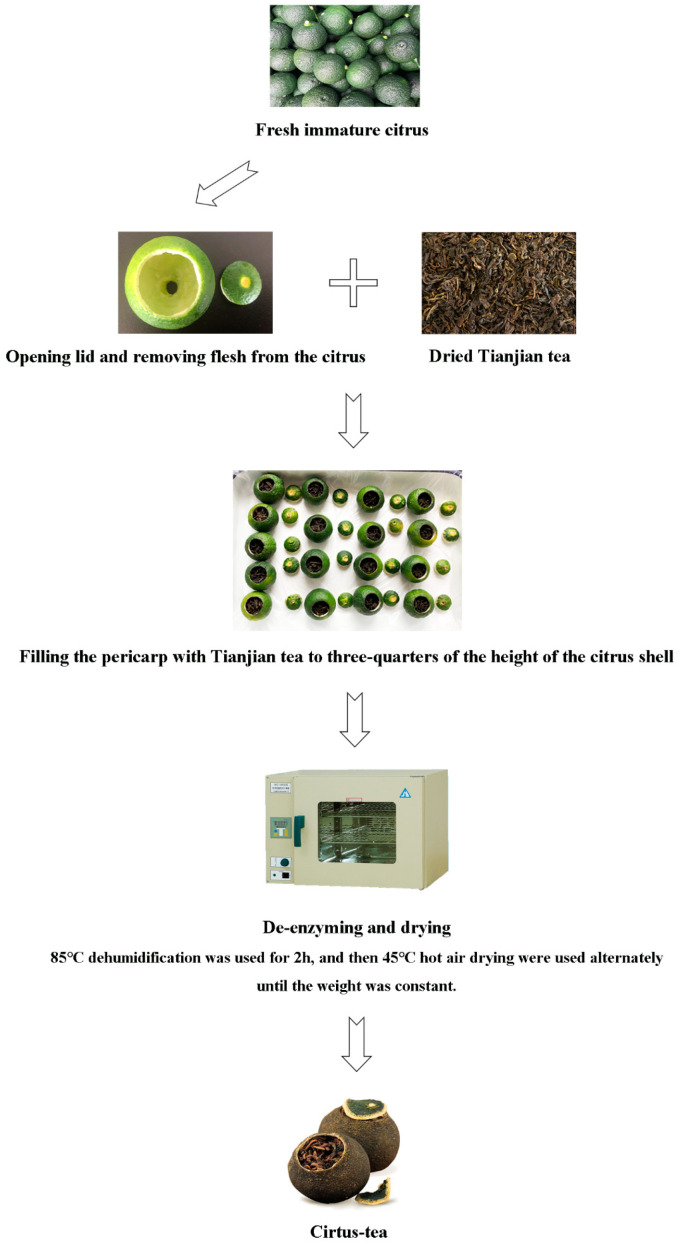
Manufacturing process of citrus-tea.

**Figure 9:**
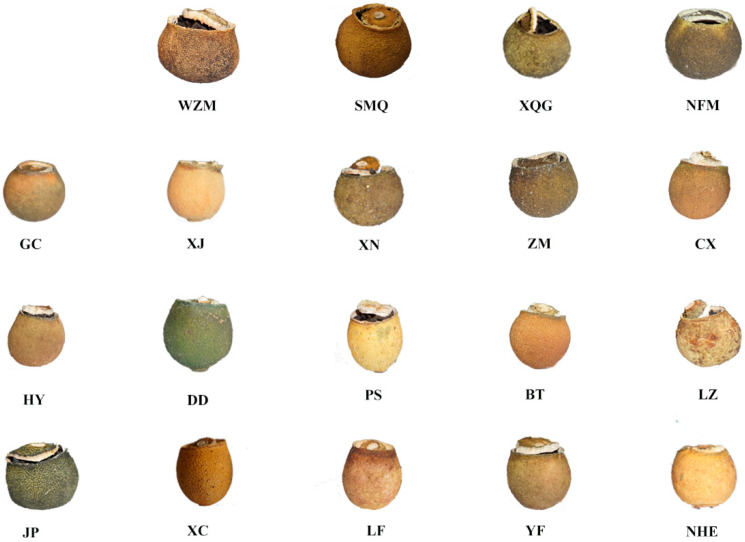
Samples of 19 types of citrus-tea.

The abbreviations and ingredient information of citrus tea are shown in Table 2. The sample processing flowchart is shown in Figure 8. Briefly, a 2-cm-diameter hole was created at the top of the citrus fruit, and the flesh was removed entirely with a spoon through the hole. Then, Tianjian tea was filled to three-quarters of the height of the citrus shell. Thereafter, it was inactivated at 85 °C for 2 h and then dried at 45 °C until a constant weight was obtained. The samples were divided into 19 groups. Figure 9 shows images of the groups.

After lapping with liquid nitrogen, each type of citrus tea was ground with a multifunctional grinder and passed through a 300-mesh filter to form powdered samples. Then, they were placed inside a tin foil bag, sealed and stored at −18 °C for further analysis.

### 3.3. HS-GC-IMS Analysis Method

The VOCs in citrus tea samples were analyzed using the FlavourSpec^®^ HS-GC-IMS instrument (the G.A.S. Department of Shandong HaiNeng Science Instrument Co., Ltd., Shandong, China) following Denawaka’s method [54]. The combined equipment includes an Agilent 490 gas chromatograph (Agilent Technology, Palo Alto, CA, USA) equipped with an FS-SE-54-CB-1 capillary column (15 cm × 0.53 mm), IMS apparatus (FlavourSpec^®^, Gesellschaft für analytische Sensorsysteme mbH, Dortmund, Germany) and an autosampler unit (CTC Analysis AG, Zwingen, Switzerland). In brief, 1.00 g of fully ground sample was placed into a 20 mL headspace glass sampling bottle and incubated at 80 °C for 20 min. After incubation, 100 μL of headspace was automatically injected into the syringe (85 °C, no split mode) with a heated syringe at 45 °C and then driven by nitrogen into a 60 °C capillary column. The specific process was as follows: 2 mL/min held for 20 min and 100 mL/min lasting for 10 min. The speed of drift gas (nitrogen) was set to 150 mL/min. The analytes were driven to the ionization chamber to be ionized in positive ion mode by a 3H ionization source of 300 MBq activities. The resulting ions were driven to the drift tube (9.8 cm in length), which was operated at 45 °C and 5 kV of voltage. The drift gas (nitrogen gas) was set at 150 mL/min, and each spectrum had an average of 12 scans. N-ketones C4-C9 (Sinopharm Chemical Reagent Beijing Co., Ltd., Beijing, China) were used as external references to calculate the RI of volatile compounds. Volatile compounds were identified by comparing the RI and drift time (the time it takes for ions to reach the collector through the drift tube, in milliseconds) of the standard in the GC-IMS library.

All data were acquired in the positive ion mode and processed using the LAV software (version 2.0.0, G.A.S). The spectra were analyzed using the LAV software, and different profiles and fingerprints of volatile components were constructed using the reporter and gallery plug-ins. The NIST and IMS databases were built into the software for qualitative analysis of the materials.

### 3.4. HS-SPME-GCMS Analysis Method

VOCs were extracted by solid-phase microextraction (SPME) using divinylbenzene/polydimethylsiloxane (65 and 50/30 μm) (Supelco, Bellefonte, PA, USA) in accordance with Lu’s method with slight modifications [55]. The SPME fiber was conditioned at 250 °C for 15 min prior to use. Firstly, 6.00 g of fine ground sample (the same sample mentioned in Section 3.3) was weighed into a 20 mL glass vial with 4 mL saturated salt solutions of NaCl (Sangon Biotech, Shanghai, China) and 10 μL cyclohexane (50 mg/L, ethanol diluted) as the internal standard. Thereafter, the vials were immersed immediately in a water bath (60 °C) for 5 min of balance, and the SPME fiber was then pushed into the headspace of the vial for extracting the volatile compounds at 60 °C for 30 min. The analytes were finally desorbed for 5.5 min at 250 °C in the gas chromatograph injector in splitless mode.

GC-MS analysis was performed on an Agilent 6890N-5973 GC-MS instrument (Agilent Technologies Inc., Santa Clara, CA, USA) with an Agilent 19091S-433 DB-5MS capillary column (30 m × 250 μm × 0.25 μm, Agilent Technologies Inc., Santa Clara, CA, USA) in accordance with the method described by Bi et al. [56]. The initial oven temperature was 50 °C, which was held for 5 min, increased at 3 °C/min to 125 °C and then increased to 180 °C at 2 °C/min. Finally, it was increased to 230 °C at 15 °C/min and held for 6 min. The carrier gas was pure helium (99.999%) at 1 mL/min. A mass detector was used in electron impact mode at 70 eV, and the ion source temperature was 230 °C. The mass spectra were scanned from 35 to 550 amu. The volatile components were tentatively identified by comparing the mass spectra and RI in the standard NIST 08 library, and the internal standard method was used to calculate the levels of volatile compounds.

The concentrations of volatile compounds were quantified on the basis of the internal standard using GC-FID [57] and calculated using the equation Cx = n × Cis, where Cx is the concentration of x compound, n is the peak area ratio of x compound/internal standard, and Cis is the internal standard concentration in the sample. The OAVs of selected volatiles were calculated by dividing the concentration with the threshold value [58].

### 3.5. Data Analysis

All assays in the manuscript were performed at least in triplicate, and the results are presented as mean ± standard deviation. OPLS-DA was conducted using SIMCA-P 14.1 (UMetrics AB, Umea, Sweden) software. One-way analysis of variance was used to analyze the OAVs of selected key volatile compounds at *P* < 0.05 through IBM SPSS Statistics 20.0 (SPSS Inc., Chicago, IL, USA). PCC was used SPSS 20.0. Cytoscape 3.7.1 was used to visualize the r value between pairs of VOCs.

## 4. Conclusions

In general, the study explored the 378 aromas of 19 citrus teas by using HS-GC-IMS and HS-SPME-GC-MS. Amongst them, the content of hydrocarbons accounted for a large proportion of most citrus teas, followed by alcohols, esters, ketones and aldehydes. In addition, a few benzene derivatives, nitrogen compounds and phenolic acids were identified. The fingerprint of HS-GC-IMS could be obtained directly and easily to facilitate the comparison of VOCs between different varieties, so the aroma characteristics of citrus tea can be rapidly identified and traced. IMS was confirmed by OPLS-DA to be a complementary analytical screening technique for citrus tea. Furthermore, 68 aroma components (OAV > 1) indicated that they significantly contributed to the overall aroma of citrus tea. Odorants such as decanal, *β*-lonone, *β*-ionone, *β*-myrcene and *D*-limonene also contributed significantly to all samples. Moreover, special aromatic biomarkers were found in certain citrus teas. For example, ethyl caprate and methyl caprate were only detected in citrus tea prepared by ZM; citral exists in the VOCs of BT. According to PCA, the samples of different varieties were well separated. Finally, through PCC analysis, the correlation of VOCs amongst different samples could be determined, providing a reference for subsequent analysis. Hence, the results can be used as a basis for evaluating the quality and tracing the origins of citrus tea from different production areas and resources.

## Figures and Tables

**Table 1:** Odor detection thresholds (ODT) and odor activity values (OAV) of major odor active compounds in citrus-tea as detected by headspace solid-phase microextraction-gas chromatography-mass spectrometry (HS-SPME-GC-MS).

No	Compound	Odor Quality	ODT ^a^ (μg /kg)	OAV ^b^																		
				WZM	SMQ	XQG	NFM	GC	XJ	XN	ZM	CX	HY	DD	PS	LZ	BT	JP	XC	LF	YF	NHE
Phenolic acids																						
A1	Acetic acid	Vinegar	99,000	<0.2	<0.2	<0.2	<0.2	<0.2	<0.2	<0.2	<0.2	<0.2	<0.2	<0.2	<0.2	<0.2	<0.2	<0.2	<0.2	<0.2	<0.2	<0.2
A2	Tridecanoic acid	Vinegar	10,000	–	0.3	–	–	–	–	–	–	–	–	–	–	–	–	–	–	–	–	–
A3	n-Decanoic acid	Oily	10,000	–	–	–	–	–	–	–	<0.2	–	–	–	–	–	–	–	–	–	–	–
A4	Thymol	Thyme, spiced	1700	–	–	–	–	2	–	–	–	1	–	–	–	3	–	1	–	–	–	–
A5	Carvacrol	Herbal	2290	16	7	4	2	10	19	5	3	12	11	7	8	2	10	1	27	3	1	11
Esters																						
B1	Linalyl acetate	Petitgrain	1000	49	35	–	–	–	–	37	–	–	–	240	–	–	26	–	41.16	–	44.914	–
B2	Methyl benzoate	Floral, fruity	73	–	–	–	–	–	304	–	–	–	–	–	–	394	–	–	–	–	–	–
B3	Ethyl caprate	Coconut	5	–	–	–	–	–	–	–	202	–	–	–	–	–	–	–	–	–	–	–
B4	Octyl acetate	Wax	47	137	63	–	100	–	–	81	66	93	66	308	67	–	–	–	98	54	95	–
B5	Geranyl acetate	Rose, floral	150	117	101	127	154	413	48	97	55	100	87	625	71	273	94	98	104	67	130	195
B6	Decyl acetate	Wax, honey	5900	–	0.8	–	–	0.8	–	–	–	–	–	–	–	–	–	<0.2	–	–	–	–
B7	1-Decanol acetate	Orange, rose, ananas	225	–	–	–	–	–	–	–	3	7	4	45	5	–	–	–	–	–	–	–
B8	Methyl caprate	Nicotian	4.3	–	–	–	–	–	–	–	113	–	–	–	–	–	–	–	–	–	–	–
Alcohols																						
C1	Terpinen-4-ol	Woody	1200	18	–	–	–	–	16	24	–	14	10	26	10	40	–	30	15	–	–	16
C2	1-Octanol	Citrus, sweet, oily	125.8	–	35	157	86	–	52	65	37	–	–	–	–	–	–	–	–	38	57	–
C3	Linalool	Floral, fruity, sweet	0.22	658,386	2,103,090	674,931	1,225,683	836,653	850,477	780,538	5,380,078	598,228	1,326,022	426,628	488,540	1,315,688	791,603	415,056	988,041	1,755,751	492,007	672,381
C4	Nerolidolcistrans	Malic, rose, woody	2250	–	–	–	–	–	–	–	–	–	–	–	–	1	–	–	–	–	–	–
C5	p-Menth-1-en-8-ol	Lemon, minty, piney	1200	12	33	713	46	–	19	26	23	12	8	41	9	43	9	29	13	–	19	–
C6	Nerol	Citrus, lemon, minty	680	–	–	–	41	–	–	38	–	25	–	–	20	–	–	–	–	38	48	–
C7	(Z)-Carveol	Citrus, spearmint	250	159	73	375	149	519	232	148	62	80	106	315	98	535	171	121	178	46	189	236
C8	(E)-Carveol	Citrus, spicy	250	–	43	–	–	197	71	–	–	–	–	–	–	–	33	52	–	–	–	–
C9	*L*(−)-Perillyl alcohol	Grassy, woody, floral	7000	–	–	4	2	–	1	–	–	–	–	–	–	6	–	–	–	–	–	–
C10	Elemol	Grassy, floral	100	5	43	16	–	7	6	4	–	24	4	–	4	–	–	22	–	–	–	7
C11	Phytol	Vegetal	640	–	–	–	0.5	–	–	0.3	0.6	–	–	–	–	–	–	–	–	–	–	0.5
C12	*trans*-Nerolidol	Malic, rose	250	–	–	–	–	–	–	–	–	8	–	126	–	–	–	–	–	–	–	–
C13	(+)-*β*-Citronellol	Rose	40	–	–	1138	–	–	–	–	576	–	–	–	–	–	–	–	–	–	–	–
C14	*L*-*α*-Terpineol	Lilac	9180	–	–	–	–	5	–	–	–	–	–	–	–	–	–	–	–	–	–	–
C15	*L*(−)-Menthol	Minty	2280	–	–	3	–	–	–	–	–	–	–	–	–	–	–	–	–	–	–	–
C16	2-heptanol	Potato, cheese, milk, powder	65	–	–	–	–	–	–	–	–	–	–	–	2	–	–	–	–	–	–	–
C17	(±)-*trans*-4-Thujanol	Minty, green	55,000	–	–	–	–	–	–	–	<0.2	–	–	–	–	–	–	–	–	–	–	–
C18	cis-Linaloloxide	Woody, floral	100	–	–	–	–	–	–	–	–	–	–	25	–	–	–	–	–	–	–	–
Aldehydes																						
D1	Dodecanal	Lilac, violet	10	–	–	–	–	–	–	–	–	–	–	–	–	–	70	–	–	60	–	–
D2	Benzeneacetaldehyde	Malic	6.3	–	–	–	–	–	647	–	–	–	–	–	–	–	–	–	–	–	–	–
D3	Nonanal	Citrus, green, fruity	1.1	–	–	–	92,371	–	–	–	–	–	–	–	–	–	–	–	–	–	–	–
D4	Citronellal	Floral, rose, sweet	6	–	158	–	–	–	–	–	375	–	–	–	–	–	–	–	–	–	–	–
D5	Citral	citric	32	–	–	–	–	–	–	–	–	–	–	–	–	–	65	–	–	–	–	–
D6	Undecanal	Wax, floral	12.5	–	–	–	–	–	–	–	–	904	41	–	31	–	–	–	–	–	139	145
D7	Octanal	citrusy, soapy	0.587	–	–	7234	25,880	–	–	–	8074	–	–	–	–	–	–	–	–	–	–	–
D8	Decanal	Citrus, fatty, green	3	2088	2420	6087	9097	7563	1102	3487	8960	2202	1829	6956	1448	4375	2412	1893	2336	2365	6635	4346
D9	(S)-(−)-Perillaldehyde	Green, oily, fatty, cherry	30	–	233	578	–	–	–	–	–	–	–	–	–	–	–	–	–	–	–	–
D10	(E)-2-Decenal	Floral, sweet	17	–	–	129	–	–	–	–	–	–	–	–	–	–	–	–	–	–	–	–
D11	*L*-Perillaldehyde	Perilla-like, spicy	30	136	–	–	550	498	176	329	205	164.741	76	589	94	580	138	162	172	125	376	318
D12	*α*-Sinensal	orange	220	–	–	50	–	–	–	3	3	–	–	–	–	–	–	–	–	–	–	–
D13	Tridecanal	Citrus, wax, oily	10	–	–	189	–	988	–	–	–	–	–	–	–	–	–	–	–	–	–	–
D14	Benzaldehyde	Fruity, almond	750.89	–	–	–	–	–	–	–	–	–	–	0.5	–	–	–	–	–	–	–	–
D15	(+)-Citronellal	Lemon, citronella, rose	30	–	–	–	91	–	–	–	–	–	–	–	–	–	–	–	–	–	–	–
Ketones																						
E1	(+)-Dihydrocarvone	Herbs	3250	1.822	–	2.291	–	–	–	–	–	0.7	–	–	–	–	–	–	2	–	–	–
E2	Hydroxyacetone	Tobacco-like	80,000	–	–	–	–	–	–	–	<0.2	–	–	–	–	–	–	–	–	–	–	–
E3	*L*(−)-Carvone	Carvi	7	–	–	–	–	–	–	–	–	–	–	–	–	–	–	–	–	36	–	–
E4	*D*(+)-Carvone	Minty, minty, licorice	160	144	979	295	158	614	208	1511	91	145	82	383	117	578	153	143	209	–	195	186
E5	Piperiton	Minty	680	–	–	2	1	–	–	1	1	–	–	–	–	–	1	2	–	–	2	–
E6	*β*-Lonone	Violet	0.007	–	–	–	–	–	–	–	183,529	–	–	–	233,159	504,179	–	–	202,171	–	457,431	–
E7	*β*-Ionone	Woody, violet	3.5	–	–	559,318	1145	899,822	–	798	–	572	–	–	–	–	463	–	–	347	–	534,380
E8	*α*-Ionone	Woody, violet	3.78	–	223	–	129	–	–	–	–	–	–	–	66	–	–	–	–	–	–	–
E9	4-Methylacetophenone	Hawthorn-like	21	–	–	–	–	686	–	–	–	–	–	–	–	–	–	–	–	–	–	–
E10	(E,E)-3,5-Octadien-2-one	Floral	100	–	–	–	–	–	–	–	–	29	–	–	–	–	–	26	–	–	–	–
E11	6-Methylhept-5-en-one	Citronnelle	68	32	–	–	–	–	–	11	–	–	–	–	1	–	2	–	–	–	21	–
E12	3-Hydroxy-2,3-dihydromaltol	Floral	35,000	–	–	–	–	–	–	–	<0.2	–	–	–	–	–	–	–	–	–	–	–
Hydrocarbons																						
F1	1r-.*α*.-Pinene	Pine-like	2.2	–	–	–	–	–	–	–	3350	–	–	–	646	–	–	3318	2866	–	–	390
F2	1s-.*α*.-Pinene	Pine-like	100	–	–	–	–	–	–	–	–	–	–	15	–	–	14	–	–	–	–	–
F3	Sabinene	woody	980	–	–	9	–	–	8	7	–	–	–	–	2	1	3	–	8	–	–	12
F4	(1S)-(−)-*β*-Pinene	Resinous, pine-like	4160	–	2	–	4	0.5	–	–	–	–	–	0.5	–	–	–	–	–	–	–	–
F5	*α*-Pinene	Resin, pine, ethereal	14	218	235	628	557	465	237	22	916	414	257	72	62	160	46	72	132	234	198	48
F6	*β*-Pinene	Resinous, pungent, green, pine-like	140	64	–	–	–	–	–	–	–	35	–	–	–	–	–	56	–	–	–	–
F7	*β*-Myrcene	Tea, Ethereal, oily	1.2	24,346	19,068	14,497	29,658	21,805	16,356	14,657	14,412	20,180	14,436	46,253	9689	26,693	10,799	21,372	24,909	11,581	19,112	25,413
F8	*D*-Limonene	Citrus, lemon, minty	34	23,633	22,203	22,919	26,510	46,910	24,795	17,967	14,990	19,839	12,935	39,124	10,875	59,930	14,492	27,900	28,664	12,074	25,152	31,450
F9	(Z)-*β*-Ocimene	Green, floral, neroli	34	164	93	263	–	230	–	100	85	–	91	496	58	187	–	–	122	118	93	35
F10	*γ*-Terpinene	Sweet, citrus	1000	62	109	34	76	68	21	45	61	65	36	20	12	38	17	97	25	8	28	28
F11	Terpinolene	Resinous, pine	200	–	34	–	–	–	–	–	–	3	–	–	–	–	–	–	–	–	–	–
F12	α-Humulene	Woody, spicy	160	–	–	–	–	–	–	–	–	–	–	134	–	–	–	–	–	9	–	–
F13	*β*-Caryophyllene	Spicy, citrus	64	111	203	140	132	235	44	79	68	179	–	683	117	219	65	370	51	42	101	148
F14	*α*-Terpinene	Citrus	80	–	–	–	–	–	–	–	–	–	3	–	–	–	–	7	–	–	–	–
F15	7,11-Dimethyl-3-methylene-1,6,10-dodecatriene	Rose, sweet	87	–	–	–	–	120	–	–	–	–	–	–	–	–	–	–	–	–	–	–
F16	*β*-Phellandrene	Citrus, pepper	500	–	–	–	–	–	–	–	22	–	12	–	–	–	–	–	–	–	–	–
F17	Limonene-1,2-epoxide	Lemon-like	100	–	–	–	–	–	1	–	–	–	–	–	–	–	–	–	–	–	–	–
Others																						
G1	4-Ethylguaiacol	Sweet, spicy, herbs	89.25	–	–	–	–	130	–	–	–	–	–	–	–	–	–	–	–	–	–	–
G2	2-Methoxy-4-vinylphenol	Strong spicy	12.02	–	–	186	–	16,135	2131	–	256	204	–	–	123	9466	–	–	–	–	–	–
G3	1-Furfurylpyrrole	Filbert, coffee-like	100	–	–	–	–	–	90	–	–	–	–	–	–	181	–	–	–	–	–	–
G4	2-Propanamine	Ammonia	5000	–	–	–	–	–	–	–	–	<0.2	–	–	–	–	–	–	–	–	–	–

^a^ The thresholds of compounds in water refer to [38]. ^b^ Odor activity value: ratio of odorant concentration in the citrus tea beverage to ODT in water.

**Table 2:** Citrus tea information sheet.

Abbreviation of Citrus-Tea	Common Name of Citrus	Plant Species of Citrus	Source of Citrus	GPS Coordinate	Plucking Time	Dark Tea	Category
WZM	Wenzhou Tangerine Dafen No. 4	*Citrus unshiu Marc*	Xiangxi Autonomous Prefecture	28°27′ N, 110°17′ E	15 July 2019	Tianjian tea	Mandarin orange
SMQ	Shimeng orange	* Citrus reticulata* cv. “Shimeng”	Changde City	25°27′ N, 121°58′ E	8 July 2019		
XQG	Xiaoqing orange	*Citrus reticulata* cv. “Xiaoqing”	Xiangxi Autonomous Prefecture	28°24′ N, 110°00′ E	15 August 2019		
NFM	Nanfeng orange	*Citrus reticulata Blanco* “Kinokuni”	Xiangxi Autonomous Prefecture	28°27′ N, 110°17′ E	15 July 2019		
GC	Gongchuan orange	*Citrus unshiu* “Miyagawa Wase”	Hunan Horticultural Research Institute, Changsha	28°21′ N, 113°10′ E	18 July 2019		
XJ	Xinjing orange	*Citrus unshiu* “Xinjing”	Hunan Horticultural Research Institute, Changsha	28°21′ N, 113°10′ E	18 July 2019		
XN	Xinnv Ponkan	*Citrus reticulata Blanco* cv. *Ponkan*	Xiangxi Autonomous Prefecture	28°27′ N, 110°17′ E	20 July 2019		Ponkan
ZM	Zaomi Ponkan	*Citrus reticulata Blanco* cv. *Ponkan*	Xiangxi Autonomous Prefecture	28°27′ N, 110°17′ E	20 July 2019		
CX	Chunxiang mixed Citrus	*Citrus poonensis Hort.* ex *Tanaka*	Xiangxi Autonomous Prefecture	28°27′ N, 110°17′ E	18 July 2019		Hybrid citrus
HY	Huyou	*Citrus maxima (Burm) Merr.*	Yueyang City	29°36′ N, 113°14′ E	1 August 2019		Grapefruits
DD	Daidai lime	*Citrus aurantium* L. “Daidai”	Lianyuan city	27°69′ N, 111°67′ E	15 August 2019		Sour orange
PS	Pushi Cheng	*Citrus sinensis* (L.) *Osbeck* “Pushi Cheng”	Xiangxi Autonomous Prefecture	28°16′ N, 109°99′ E	18 August 2019		Sweet orange
BT	Bingtang orange	*Citrus sinensis* (L.) *Osbeck* “Bingtang”	Xiangxi Autonomous Prefecture	28°16′ N, 109°99′ E	18 August 2019		
LZ	Luzai honey orange	*Citrus sinensis* (L.) *Osbeck* “Luzai”	Xiangxi Autonomous Prefecture	28°27′ N, 110°17′ E	18 August 2019		
JP	Jinpenyou	*Citrus junos Sieb.* ex *Tanaka* “yuzu”	Yueyang City	29°15′ N, 113°12′ E	17 August 2019		Yuzu
XC	Tarocco blood orange	*Citrus sinensis* (L.) *Osbeck* “Tarocco”	Xiangxi Autonomous Prefecture	28°27′ N, 110°17′ E	18 August 2019		Navel orange
LF	Langfeng navel orange	*Citrus sinensis Osbeck* “Langfeng”	Shaoyang City	39°90′ N, 116°52′ E	15 September 2019		
YF	Yuanfeng navel orange	*Citrus sinensis Osbeck* “Yuanfeng”	Hunan Horticultural Research Institute, Changsha	28°19′ N, 113°14′ E	17 August 2019		
NHE	Newhall navel orange	*Citrus sinensis Osbeck* “Newhall”	Hunan Horticultural Research Institute, Changsha	28°21′ N, 113°10′ E	17 August 2019		

## Supplementary Materials

The following are available online, Figure S1: Ion migration spectra of WZM. The numbers are the identified volatile components. Figure S2: Similarity between samples of citrus-tea; the larger the dot, the higher the similarity. Figure S3: Four parts of the screenshots from the Gallery Plot fingerprints. Eleven kinds of identified peaks (a); 22 kinds of identified peaks (b); 9 kinds of identified peaks (c); 17 kinds of identified peaks (d). Table S1: Information on the identified compounds of citrus-tea by HS-GC-IMS. Table S2: Information on the quantitated compounds of citrus-tea by HS-SPME-GC-MS.

## Abbreviations

HS-GC-IMS	Headspace gas chromatography-ion mobility spectrometry
HS-SPME-GC-MS	Headspace solid-phase microextraction-gas chromatography-mass spectrometry
VOCs	Volatile organic compounds
RI	Retention index
WZM	Citrus-tea made from Wenzhou Tangerine Dafen No. 4 and Tianjian tea
SMQ	Citrus-tea made from Shimeng orange and Tianjian tea
XQG	Citrus-tea made from Xiaoqing orange and Tianjian tea
NFM	Citrus-tea made from Nanfeng orange and Tianjian tea
GC	Citrus-tea made from Gongchuan orange and Tianjian tea
XJ	Citrus-tea made from Xinjing orange and Tianjian tea
XN	Citrus-tea made from Xinnv Ponkan and Tianjian tea
ZM	Citrus-tea made from Zaomi Ponkan and Tianjian tea
CX	Citrus-tea made from Chunxiang mixed Citrus and Tianjian tea
HY	Citrus-tea made from Huyou and Tianjian tea
DD	Citrus-tea made from Daidai lime and Tianjian tea
PS	Citrus-tea made from Pushi Cheng and Tianjian tea
BT	Citrus-tea made from Bingtang orange and Tianjian tea
LZ	Citrus-tea made from Luzai honey orange and Tianjian tea
JP	Citrus-tea made from Jinpenyou and Tianjian tea
XC	Citrus-tea made from Tarocco blood orange and Tianjian tea
LF	Citrus-tea made from Langfeng navel orange and Tianjian tea
YF	Citrus-tea made from Yuanfeng navel orange and Tianjian tea
NHE	Citrus-tea made from Newhall navel orange and Tianjian tea
PCA	Principal component analysis
OPLS-DA	Orthogonal partial least squares discrimination analysis
OAV	Odor activity value
ODT	Odor detection thresholds
VIP	Variable importance for the projection
PCC	Pearson correlation coefficients
