# Supplementary material for: Characteristic Volatile Fingerprints and Odor Activity Values in Different Citrus-Tea by HS-GC-IMS and HS-SPME-GC-MS
Source: Molecules. 2020 Dec 19;25(24):6027. doi: 10.3390/molecules25246027 (PMC7766395; doi:10.3390/molecules25246027)
Supplement: Supplementary file 1 [file molecules-25-06027-s001.pdf]

Supplementary Materials

Table S1. Information on the identified compounds of citrus-tea by HS-GC-IMS.

NO.	Compound	CAS#	Formula	MW[a]	RI[b]	Rt[c] (sec)	Dt[d] [RIPrel]	Comment
1	γ-Decalactone	C706149	$C_{10}H_{18}O_2$	170.3	1538.1	1169.307	1.4715	
2	Decanal	C112312	$C_{10}H_{20}O$	156.3	1260.2	737.434	1.543	Monomer
3	Decanal	C112312	$C_{10}H_{20}O$	156.3	1259.4	736.205	2.056	Dimer
4	Phenylethyl acetate	C103457	$C_{10}H_{12}O_2$	164.2	1318.7	828.421	1.3119	
5	Phenylacetic acid	C103822	$C_8H_8O_2$	136.1	1283.9	774.321	1.3301	
6	α-Terpineol	C98555	$C_{10}H_{18}O$	154.3	1192.7	632.58	1.2204	
7	β-Citronellol	C106229	$C_{10}H_{20}O$	156.3	1229.6	689.918	1.342	
8	Linalool	C78706	$C_{10}H_{18}O$	154.3	1101.4	490.694	1.2217	
9	γ-Terpinene	C99854	$C_{10}H_{16}$	136.2	1077.7	453.848	1.2194	
10	β-Ocimene	C13877913	$C_{10}H_{16}$	136.2	1051.7	414.628	1.2156	Monomer
11	β-Ocimene	C13877913	$C_{10}H_{16}$	136.2	1051.3	413.995	1.7034	Dimer
12	Limonene	C138863	$C_{10}H_{16}$	136.2	1023.4	374.774	1.2979	Monomer
13	Limonene	C138863	$C_{10}H_{16}$	136.2	1022.9	374.142	1.7148	Dimer
14	Myrcene	C123353	$C_{10}H_{16}$	136.2	991.3	335.554	1.7186	
15	β-Pinene	C127913	$C_{10}H_{16}$	136.2	975.0	318.474	1.2181	Monomer
16	β-Pinene	C127913	$C_{10}H_{16}$	136.2	973.7	317.209	1.6375	Dimer
17	Camphene	C79925	$C_{10}H_{16}$	136.2	946.2	292.538	1.2131	
18	α-Pinene	C80568	$C_{10}H_{16}$	136.2	927.2	277.988	1.6667	
19	Octanal	C124130	$C_8H_{16}O$	128.2	1006.6	353.266	1.4069	Monomer
20	Octanal	C124130	$C_8H_{16}O$	128.2	1007.1	353.899	1.8263	Dimer
21	2-Ethyl-6-methylpyrazine	C13925036	$C_7H_{10}N_2$	122.2	999.7	345.043	1.1776	
22	Benzaldehyde	C100527	C_7H_6O	106.1	960.9	305.189	1.1522	Monomer
23	Benzaldehyde	C100527	C_7H_6O	106.1	963.0	307.087	1.4728	Dimer
24	2-Acetylfuran	C1192627	$C_6H_6O_2$	110.1	916.1	270.092	1.117	
25	Heptanal	C111717	$C_7H_{14}O$	114.2	903.7	261.892	1.3342	Monomer
26	Heptanal	C111717	$C_7H_{14}O$	114.2	902.9	261.306	1.6987	Dimer
27	Cyclohexanone	C108941	$C_6H_{10}O$	98.1	899.6	259.256	1.1485	Monomer

28	Cyclohexanone	C108941	$C_6H_{10}O$	98.1	896.8	257.499	1.4557	Dimer
29	1-Hexanol	C111273	$C_6H_{14}O$	102.2	875.1	244.613	1.3274	
30	(E)-2-Hexenol	C928950	$C_6H_{12}O$	100.2	852.2	232.02	1.1834	Monomer
31	(E)-2-Hexenol	C928950	$C_6H_{12}O$	100.2	853.9	232.898	1.5153	Dimer
32	Furfurol	C98011	$C_5H_4O_2$	96.1	831.0	221.184	1.0855	Monomer
33	Furfurol	C98011	$C_5H_4O_2$	96.1	830.4	220.891	1.3342	Dimer
34	Hexanal	C66251	$C_6H_{12}O$	100.2	795.6	204.49	1.2599	Monomer
35	Hexanal	C66251	$C_6H_{12}O$	100.2	794.4	203.905	1.5626	Dimer
36	2-Hexanone	C591786	$C_6H_{12}O$	100.2	784.1	199.33	1.1898	Monomer
37	2-Hexanone	C591786	$C_6H_{12}O$	100.2	783.4	199.017	1.5053	Dimer
38	1-Pentanol	C71410	$C_5H_{12}O$	88.1	765.7	191.364	1.2533	

Table S1. (continued).

NO.	Compound	CAS#	Formula	MW[a]	RI[b]	Rt[c] (sec)	Dt[d] [RIPrel]	Comment
39	Dimethyl disulfide	C624920	$C_2H_6S_2$	94.2	737.6	179.961	0.9877	
40	Acetoin	C513860	$C_4H_8O_2$	88.1	717.6	172.62	1.0604	Monomer
41	Acetoin	C513860	$C_4H_8O_2$	88.1	713.9	171.371	1.3302	Dimer
42	2-Ethylfuran	C3208160	C_6H_8O	96.1	689.7	163.717	1.3116	
43	Pentanal	C110623	$C_5H_{10}O$	86.1	700.0	166.841	1.4241	
44	2-Pentanone	C107879	$C_5H_{10}O$	86.1	698.1	166.216	1.3666	
45	2-Methylbutanal	C96173	$C_5H_{10}O$	86.1	668.1	157.938	1.4013	
46	3-Methylbutanal	C590863	$C_5H_{10}O$	86.1	651.7	154.033	1.1991	Monomer
47	3-Methylbutanal	C590863	$C_5H_{10}O$	86.1	653.1	154.345	1.4063	Dimer
48	Ethyl acetate	C141786	$C_4H_8O_2$	88.1	609.7	145.286	1.0977	Monomer
49	Ethyl acetate	C141786	$C_4H_8O_2$	88.1	610.4	145.442	1.337	Dimer
50	Acetic acid	C64197	$C_2H_4O_2$	60.1	630.0	149.347	1.0494	
51	Butanal	C123728	C_4H_8O	72.1	603.2	144.036	1.2905	
52	2-Butanone	C78933	C_4H_8O	72.1	590.2	141.537	1.2465	
53	2,3-Butanedione	C431038	$C_4H_6O_2$	86.1	562.4	136.227	1.167	
54	Acetone	C67641	C_3H_6O	58.1	484.8	121.388	1.1154	
55	Ethanol	C64175	C_2H_6O	46.1	452.2	115.14	1.0469	
56	2-Furfurylthiol	C98022	C_5H_6OS	114.2	908.8	265.25	1.1008	

57	Methyl N-Methyl-anthranilate	C85916	$C_9H_{11}NO_2$	165.2	1510.4	1126.314	1.2664
58	Geraniol	C106241	$C_{10}H_{18}O$	154.3	1369.8	907.809	1.2201
59	Methylpropanal	C78842	C_4H_8O	72.1	554.4	134.703	1.2832

[a]MW: molecular mass; [b]RI: retention index; [c]Rt: retention time; [d]Dt: drift time.

Table S2. Information on the quantitated compounds of citrus-tea by HS-SPME-GC-MS.

No.	Compound	Concentration (μg /g)																		
		**WZM**	**SMQ**	**XQG**	**NFM**	**GC**	**XJ**	**XN**	**ZM**	**CX**	**HY**	**DD**	**PS**	**LZ**	**BT**	**JP**	**XC**	**LF**	**YF**	**NHE**
	**Phenolic_acids**																			
1	d-Alanine	-	-	-	-	1.04±0.22	0.35±0.01	-	-	-	-	6.53±0.27	-	-	4.41±0.46	-	-	-	-	-
2	Acetic acid	0.47±0.03	0.64±0.09	0.46±0.01	0.76±0.06	3.38±0.32	0.38±0.04	0.75±0.06	0.64±0.03	0.33±0.02	0.47±0.07	1.97±0.04	1.08±0.32	0.36±0.02	0.74±0.06	0.12±0.01	1.13±0.16	0.74±0.05	1.24±0.64	1.64±0.13
3	dl-Alanine	-	-	-	-	0.50±0.03	1.36±0.27	-	-	-	-	-	-	-	-	-	-	-	-	-
4	(2,5-dioxo-1-pyridin-3-ylmethyl-4-trifluoromethylimidazolidin-4-yl)amide-Cyclopropanecarboxylic acid	-	-	-	4.76±0.64	-	-	-	-	-	-	-	-	-	-	-	-	-	-	-
5	Tridecanoic acid	-	2.73±0.29	-	-	-	-	-	-	-	-	-	-	-	-	-	-	-	-	-
6	n-Decanoic acid	-	-	-	-	-	-	-	1.03±0.36	-	-	-	-	-	-	-	-	-	-	-
7	n-Hexadecanoic acid	-	-	-	-	-	-	-	0.90±0.02	-	-	-	-	-	-	-	-	-	-	-
8	3-(2-Isopropyl-5-methylphenyl)-2-methylpropionic acid	0.48±0.03	1.63±0.21	0.72±0.04	-	-	-	-	-	-	-	-	-	-	-	-	-	-	-	-
9	4-hydroxy-1H-Indole-3-carboxylic acid	-	-	-	-	-	-	-	-	-	2.31±0.31	-	-	-	-	-	-	-	-	-
10	(1R,2S)-2-Acetyl-1-methylcyclobutaneacetic acid	-	-	-	-	-	-	-	-	-	-	-	-	-	-	4.93±0.68	-	-	-	-

11	3-amino-2-methyl-Butanoic acid	-	-	-	-	-	-	-	-	-	-	-	-	-	0.69±0.04	-	-	-	-	-
12	(.+/-.)-α,4-dihydroxy-Benzeneacetic acid	-	-	-	-	-	-	-	-	-	-	-	0.56±0.06	-	-	-	-	-	-	-
13	3-amino-2-methyl-Acetic acid	-	-	-	-	-	-	-	-	-	-	-	-	-	-	-	-	0.37±0.07	-	-
14	i-Propyl tricosanoate	3.01±0.32	5.51±0.74	6.32±1.03	2.86±0.42	0.36±0.07	1.42±0.09	-	-	2.24±0.37	-	-	-	-	-	-	-	-	-	-
15	Neodihydrocarveol	-	-	-	-	-	-	-	-	-	-	-	-	-	-	-	-	1.68±0.27	2.14±0.41	-
16	Thymol	-	12.95±1.29	1.79±0.54	-	-	-	3.57±0.39	-	-	1.84±0.24	-	4.49±0.75	1.99±0.37	-	-	-	-	-	-
17	Carvacrol	16.21±1.32	8.43±0.98	27.73±3.26	162.79±6.27	42.67±3.29	5.24±0.98	21.96±3.14	11.07±0.67	6.99±1.27	6.56±0.71	18.86±1.67	4.39±0.35	2.97±0.42	23.10±2.09	62.24±4.19	6.87±1.28	3.05±0.74	24.44±3.29	25.59±2.48
	Esters																			
18	i-Propyl tricosanoate	3.01±0.65	5.51±0.42	6.32±0.68	2.86±0.37	0.36±0.02	1.42±0.13	-	-	2.24±0.14	-	-	-	-	-	-	-	-	-	-
19	Linalyl acetate	48.52±4.38	34.71±2.29	-	-	-	-	36.95±2.28	-	-	-	239.64±9.68	-	26.23±3.28	-	-	41.16±1.68	-	44.91±2.18	-
20	Methyl geraniate	0.59±0.06	-	-	0.61±0.08	-	-	0.45±0.03	0.43±0.07	0.55±0.05	0.26±0.07	-	0.47±0.09	0.63±0.02	-	-	0.87±0.12	0.65±0.06	0.80±0.04	1.03±0.13

Table S2. (continued).

No.	Compound	Concentration (μg/g)																		
		WZM	SMQ	XQG	NFM	GC	XJ	XN	ZM	CX	HY	DD	PS	LZ	BT	JP	XC	LF	YF	NHE
21	Pentafluoropropionic acid, octyl ester	-	-	-	-	-	-	-	-	-	-	-	3.92±1.03	-	-	-	-	-	-	-
22	Methyl benzoate	-	-	-	-	-	22.21±1.36	-	-	-	-	-	-	-	28.78±2.91	-	-	-	-	-
23	Ethyl caprate	-	-	-	-	-	-	-	1.01±0.02	-	-	-	-	-	-	-	-	-	-	-
24	*trans*-Carveyl acetate	-	1.17±0.15	-	-	-	-	-	-	-	-	-	-	-	-	-	-	-	-	-
25	Bicyclogermacrene	-	-	-	-	-	0.91±0.04	-	1.48±0.38	-	-	-	-	-	-	14.62±1.38	-	-	-	-
26	Octyl acetate	6.45±0.29	2.96±0.24	-	4.70±0.68	-	-	3.83±	3.09±	4.37±0.38	3.11±0.35	14.47±1.24	3.16±0.67	-	-	-	4.63±1.03	2.53±0.31	4.48±0.38	-
27	1,6-Octadien-3-ol, 3,7-dimethyl-, 2-aminobenzoate	-	-	47.43±3.24	47.81±3.68	16.35±1.26	36.84±2.57	-	27.53±2.19	30.94±2.46	45.18±3.67	-	35.92±4.26	-	99.17±6.38	56.23±4.37	-	19.71±2.61	14.65±1.38	51.06±2.17
28	Perillyl acetate	-	-	-	-	-	-	-	-	-	-	-	-	-	-	-	1.73±0.28	-	-	-
29	Geranyl acetate (Z)	16.33±2.19	12.70±1.06	15.37±1.09	21.29±1.09	54.22±9.68	47.61±3.67	13.73±2.19	8.12±1.29	21.66±2.18	13.12±0.29	70.30±6.26	10.70±1.31	14.82±0.76	37.13±2.19	12.55±1.65	19.30±1.29	17.22±2.91	20.40±1.69	29.52±3.67
30	Geranyl acetate (E)	17.48±2.31	15.16±2.61	19.09±1.26	23.09±2.19	61.93±2.94	7.16±0.75	14.61±2.19	8.32±0.98	15.01±1.06	13.06±1.06	93.77±4.28	10.72±1.03	14.10±1.36	40.98±2.97	14.68±0.98	15.60±1.09	10.04±0.38	19.46±1.59	29.20±2.04
31	Decyl acetate	-	4.90±0.12	-	-	4.76±0.39	2.39±0.17	-	-	-	-	-	-	-	-	0.30±0.02	-	-	-	-
32	1-Decanol acetate	-	-	-	-	-	-	-	0.71±0.03	1.65±0.21	0.92±0.03	10.03±0.98	1.05±0.24	-	-	-	-	-	-	-

33	3-Hydroxymandelic acid, ethyl ester, di-TMS	-	1.79± 0.25	-	-	-	-	-	-	-	-	-	-	0.18± 0.02	-	-	-	2.12± 0.12	3.93± 0.63	-
34	linalool formate	-	-	-	-	67.45 ±3.25	45.36 ±2.26	-	-	-	-	-	-	-	-	-	-	-	-	-
35	Isopropoxycarbamic acid, ethyl ester	-	-	2.09± 0.67	-	-	-	-	-	-	-	-	-	-	-	-	-	1.98± 0.19	3.53± 0.25	-
36	Myrtenyl acetate	-	-	-	-	-	-	-	-	-	-	-	-	-	-	3.45± 0.36	-	-	-	-
37	Octyl trichloroacetate	-	-	-	-	-	-	-	-	-	-	-	-	-	-	-	-	-	-	2.61± 0.57
38	Diethyl 4,4'-methylenediallophanate	-	-	-	-	-	-	0.14± 0.02	-	-	-	-	-	-	-	-	0.37± 0.02	-	-	-
39	Butanoic acid, 2,7-dimethyloct-7-en-5-yn-4-yl ester	-	-	-	-	-	-	-	-	0.46± 0.03	-	-	-	-	-	-	-	-	-	-
40	Z-Methyl geranate	-	-	-	-	-	-	-	-	-	-	-	-	-	-	-	-	0.30± 0.03	0.42± 0.08	-
41	Methyl caprate	-	-	-	-	-	-	-	0.49± 0.07	-	-	-	-	-	-	-	-	-	-	-
42	1-Octyl trifluoroacetate	-	-	-	-	-	-	-	-	-	3.64± 0.36	-	-	-	-	-	-	-	-	-
43	Methyl 5-acetyl-2-methoxybenzoate	-	-	-	-	-	-	-	-	-	-	-	-	-	-	-	0.09± 0.0002	-	-	-
44	[2-Methyl-4-(piperidine-1-sulfonyl)phenoxy]acetic acid, methyl ester	-	-	-	-	-	-	-	-	-	-	-	-	-	-	-	-	0.26± 0.01	0.72± 0.06	-

Table S2. (continued).

No.	Compound	Concentration (μg /g)																		
		WZM	SMQ	XQG	NFM	GC	XJ	XN	ZM	CX	HY	DD	PS	LZ	BT	JP	XC	LF	YF	NHE
45	5,6,7,7a-Tetrahydro-4,4,7a-trimethyl-2(4H)-benzofuranone	-	-	-	-	-	-	-	-	-	-	-	-	-	-	-	-	3.25±0.84	1.82±0.12	-
	Alcohols																			
46	*cis*-p-Mentha-2,8-dien-1-ol	23.27±1.21	-	-	-	11.65±0.68	8.31±1.03	-	-	32.09±3.28	-	-	-	-	30.28±2.16	-	-	15.26±1.18	21.35±1.38	3.63±0.15
47	Terpinen-4-ol	21.45±3.69	-	-	-	-	19.16±2.03	28.56±1.03	-	16.22±1.75	12.22±0.98	31.61±4.67	11.54±2.37	-	47.70±6.28	35.86±4.59	17.72±3.58	-	-	18.89±1.38
48	Carveol	13.77±0.27	-	3.26±0.75	-	-	-	-	-	-	7.59±1.05	-	-	-	53.50±4.68	-	-	-	-	-
49	*trans*-*β*-Santalol	-	-	-	-	-	-	-	-	-	-	-	-	-	-	-	-	-	-	-
50	1-Octanol	-	4.43±0.36	19.71±2.06	10.85±1.36	-	6.55±0.46	8.15±0.74	4.67±0.18	-	-	-	-	-	-	-	-	4.78±0.35	7.12±1.06	-
51	(R)-Lavandulol	-	-	-	-	-	-	-	-	-	-	-	-	-	-	-	-	-	-	-
52	*cis*-*β*-Terpineol	-	-	-	-	-	-	-	-	-	-	-	-	-	-	-	-	-	-	-
53	Linalool	144.84±6.98	462.68±9.68	148.48±7.15	269.65±14.02	184.06±5.19	187.10±7.64	171.72±12.35	118.36±6.54	131.61±8.46	291.72±11.21	93.86±3.64	107.48±8.49	108.24±9.54	174.15±9.37	91.31±5.37	217.37±17.62	386.27±11.03	289.45±12.13	147.92±12.32
54	*trans*-p-Mentha-2,8-dienol	-	7.61±0.38	49.42±2.74	-	14.69±2.19	23.76±1.32	-	14.34±1.28	-	-	24.21±2.37	-	-	41.39±3.18	28.36±2.19	-	-	-	-
55	*cis*-p-Menth-2,8-diene-1-ol	37.68±3.67	4.63±0.35	43.32±3.24	41.36±6.38	10.50±1.23	15.86±1.65	41.78±3.28	8.95±1.34	17.78±2.19	53.05±4.26	32.00±4.25	29.78±3.33	41.72±4.11	18.52±2.19	54.66±3.27	63.43±5.26	20.01±2.18	26.81±2.98	62.00±3.28
56	2,6-Dimethyl-1,5,7-octatrien-3-ol	-	-	-	8.04±0.32	-	3.78±0.12	7.56±0.64	3.88±0.12	4.68±0.37	8.04±0.74	-	5.75±0.23	8.60±0.57	22.17±1.32	-	11.12±0.65	5.49±0.25	9.38±1.06	10.79±0.45

57	Nerolidolcistrans	-	-	-	-	-	-	-	-	-	-	-	0.57±0.02	-	2.45±0.25	-	-	-	-	-
58	(−)-4-Terpineol	-	25.72±2.64	140.04±21.06	40.89±2.98	-	-	-	20.51±2.75	-	-	-	-	26.71±3.29	-	-	-	18.37±1.32	32.18±2.65	-
59	Isocarveol	65.61±2.38	-	97.86±5.64	-	227.36±32.61	156.28±23.24	94.40±15.32	-	-	90.49±6.57	116.82±2.19	67.66±2.19	61.51±4.68	204.25±13.26	46.19±3.29	68.69±6.36	79.09±7.16	111.96±6.34	221.85±23.12
60	p-Menth-1-en-8-ol	13.83±1.32	39.78±2.35	85.64±6.24	55.20±2.03	-	22.91±2.16	31.62±3.61	27.73±2.31	14.98±1.67	9.04±0.68	49.66±2.34	11.02±1.37	10.83±0.68	51.15±4.22	34.63±3.19	15.54±2.31	-	22.66±1.32	-
61	*cis*-*β*-Terpineol	-	-	1.91±0.32	-	-	-	-	-	-	-	-	-	0.62±0.09	-	-	-	-	-	-
62	Nerol	-	-	-	27.82±3.21	-	-	25.81±1.32	-	17.25±2.01	-	-	13.39±1.03	-	-	-	-	25.84±2.31	32.75±2.15	-
63	2-Methyl-6-methylene-octa-1,7-dien-3-ol	-	3.64±0.35	-	-	-	-	-	-	-	-	-	-	-	-	-	-	-	-	-
64	3,6-dimethoxy-9-(2-phenylethynyl)-Fluoren-9-ol	-	1.67±0.37	-	-	-	-	-	-	-	-	-	-	-	-	-	-	-	-	-
65	(Z)-Carveol	39.77±2.03	18.24±1.32	93.75±6.24	37.26±2.13	129.66±5.12	58.01±2.17	36.91±3.25	15.52±0.96	20.09±2.16	26.52±1.38	78.74±6.28	24.52±3.64	42.79±2.19	133.69±6.27	30.31±2.19	44.48±3.65	11.43±1.32	47.13±3.61	59.00±3.27

Table S2. (continued).

No.	Compound	Concentration (μg /g)																		
		**WZM**	**SMQ**	**XQG**	**NFM**	**GC**	**XJ**	**XN**	**ZM**	**CX**	**HY**	**DD**	**PS**	**LZ**	**BT**	**JP**	**XC**	**LF**	**YF**	**NHE**
66	(+)-Verbenol	-	-	-	-	-	-	-	-	-	-	-	-	62.20 ±3.64	-	-	-	-	-	-
67	2-(4a,8-Dimethyl-1,2,3,4,4a,5,6,7-octahydro-naphthalen-2-yl)-prop-2-en-1-ol	-	0.68	-	-	-	-	-	-	-	-	-	-	-	-	-	-	-	-	-
68	(E)-Carveol	-	10.85 ±1.17	-	-	49.28 ±2.27	17.81 ±1.97	-	-	-	-	-	-	8.15± 1.03	-	13.01 ±1.24	-	-	-	-
69	*L*(−)-Perillyl alcohol	-	-	24.56 ±2.08	11.52 ±2.18	-	10.48 ±0.75	-	-	-	-	-	-	-	44.21 ±4.28	-	-	-	-	-
70	Elemol	0.48± 0.12	4.31± 0.37	1.63± 0.07	-	7.67± 0.18	0.60± 0.03	0.40± 0.02	-	2.35± 0.03	0.37± 0.02	-	0.37± 0.008	-	-	2.17± 0.43	-	-	-	0.71± 0.04
71	*cis*-nerolidol	-	-	-	-	-	-	-	-	-	1.22± 0.04	-	-	-	-	4.11± 1.02	-	-	-	1.29± 0.21
72	Espatulenol	8.30± 0.27	18.48 ±2.07	-	18.90 ±1.28	23.80 ±1.74	13.59 ±2.14	-	-	6.91± 0.15	5.25± 0.31	42.87 ±3.17	7.62± 0.38	-	28.34 ±2.16	2.00± 0.31	2.21± 0.13	3.82± 1.04	5.47± 1.03	9.08± 1.23
73	(−)-Spathulenol	-	-	30.02 ±1.25	-	34.46 ±2.15	26.32 ±2.17	5.92± 0.81	1.93± 0.04	-	-	-	-	2.80± 0.06	3.27± 0.31	68.35 ±3.24	-	-	-	3.65± 0.75
74	*cis*-Lanceol	-	-	1.49± 0.21	-	-	-	-	-	-	-	-	-	-	-	-	-	-	-	-
75	6-Isopropenyl-4,8a-dimethyl-1,2,3,5,6,7,8,8a-octahydro-naphthalen-2-ol	1.29± 0.14	4.33± 0.24	-	-	5.48± 1.24	2.68± 0.38	-	-	-	-	-	-	-	-	-	-	-	-	-
76	15-Copaenol	-	-	0.94± 0.09	-	-	-	-	-	-	-	-	-	-	-	-	-	-	-	-

77	*τ*-Muurolol	-	0.98±0.21	-	-	-	-	-	-	0.38±0.03	-	-	-	-	-	-	-	-	-	-
78	*β*-Eudesmol	-	-	-	-	-	-	-	-	0.40±0.01	-	-	-	-	-	1.13±0.02	-	-	-	-
79	1-(1-methylene-2-propenyl)-Cyclo pentanol	-	-	1.38±0.36	-	-	-	-	-	-	-	-	-	-	-	-	-	-	-	-
80	Phytol	-	-	-	0.29±0.12	-	-	0.33±0.03	0.18±0.02	-	-	-	-	-	-	-	-	-	-	0.31±0.01
81	*trans*-Nerolidol	-	-	-	-	-	-	-	-	1.93±0.32	-	31.61±2.37	-	-	-	-	-	-	-	-
82	(+)-*β*-Citronellol	-	-	45.53±2.37	-	-	-	-	23.05±2.36	-	-	-	-	-	-	-	-	-	-	-
83	p-Mentha-1(7),8(10)-dien-9-ol	-	-	-	-	-	-	-	-	-	-	-	2.33±0.35	3.18±0.27	-	-	-	2.68±0.16	3.84±0.17	7.30±0.45
84	2-Methyl-1,5-heptadien-4-ol	0.64±0.03	-	-	-	-	-	0.71±0.12	-	-	-	-	-	-	-	-	-	1.32±0.31	1.14±0.01	-
85	2-(4-Methylphenyl)propan-2-ol	-	8.82±0.65	-	-	-	-	-	-	-	-	-	-	-	-	-	-	-	-	-
86	*L*-*α*-Terpineol netural	-	-	-	-	44.35	-	-	-	-	-	-	-	-	-	-	-	-	-	-
87	Nerolidol 2	-	-	-	-	-	-	-	-	-	-	-	0.83	0.65	-	-	-	-	-	-

Table S2. (continued).

	Compound	Concentration (μg/g)																		
		WZM	SMQ	XQG	NFM	GC	XJ	XN	ZM	CX	HY	DD	PS	LZ	BT	JP	XC	LF	YF	NHE
88	1-(methylenecyclopropyl)-Cyclopentanol	-	19.33 ±2.36	-	-	-	-	-	-	24.82 ±1.12	-	-	-	-	-	-	-	-	-	-
89	(2Z,6E)-Farnesol	-	-	-	-	-	-	3.84± 0.32	1.40± 0.12	-	2.34± 0.21	-	-	1.58± 0.31	-	-	-	-	-	-
90	Coolact	-	-	4.82± 0.334	-	39.99 ±3.65	43.21 ±3.29	-	-	-	-	-	-	-	-	-	-	-	-	-
91	3-Methoxybenzyl alcohol	-	-	-	-	8.11± 1.31	4.36± 0.31	-	-	-	-	-	-	-	-	-	-	-	-	-
92	1-chloro-2-Propanol	-	-	0.41± 0.03	-	-	0.26± 0.01	-	-	-	0.20± 0.009	-	-	-	-	0.30± 0.03	-	0.03± 0.0002	-	-
93	2-(2-Hydroxyethoxy)ethanol 1-nitrate	-	-	0.07± 0.0001	-	-	-	-	-	-	-	-	-	-	-	-	-	2.68± 0.68	3.78± 0.46	-
94	*L*(−)-Menthol	-	-	6.75± 0.67	-	-	-	-	-	-	-	-	-	-	-	-	-	-	-	-
95	p-Menth-1-en-9-ol(8CI)	-	-	11.62 ±2.36	-	-	-	-	-	-	-	-	-	-	-	-	-	-	-	-
96	4-Methoxyphenyl methyl carbinol	-	-	-	-	-	-	-	-	-	-	-	-	-	10.93 ±1.32	-	-	-	-	-
97	*trans*-3-methylpent-3-ene-5-ol	-	-	-	-	2.36± 0.21	6.09± 1.03	-	-	-	-	-	-	-	-	-	-	-	-	-
98	1,3-Methano-5bH-cyclobuta[cd]pentalen-5b-ol, octahydro-	-	-	-	-	4.67± 0.45	9.02± 0.97	-	-	-	-	-	-	-	-	-	-	-	-	17.19 ±2.61

99	4-methylene-Cyclohexanemethanol	-	-	-	-	-	-	-	-	-	-	-	-	-	-	-	-	-	-	1.43±0.13
100	2-Heptanol	-	-	-	-	-	-	-	-	-	-	-	0.14±0.02	-	-	-	-	-	-	-
101	4-methylene-2-Decen-1-ol	-	-	-	-	-	-	-	7.45±1.32	-	0.44±0.16	-	0.26±0.01	-	-	-	-	-	-	-
102	4-methylene-Oleyl alcohol	-	-	-	-	-	-	3.18±0.16	-	-	-	-	-	-	-	-	-	-	-	-
103	Geranyl linalool	-	-	-	-	-	-	0.65±0.07	-	-	-	-	-	-	-	-	-	-	-	-
104	E-11,13-Tetradecadien-1-ol	-	-	-	-	-	-	-	-	-	-	-	-	3.29±0.32	-	-	-	-	-	-
105	(E)-11-Tetradecen-1-ol	-	-	-	-	-	-	-	-	-	-	-	-	0.69±0.07	-	-	-	-	-	-
106	Cumicalcohol	-	-	-	-	-	-	-	-	-	0.71±0.06	-	-	-	-	-	-	1.09±0.21	0.67±0.03	-
107	(±)-*trans*-4-Thujanol	-	-	-	-	-	-	-	1.13±0.31	-	-	-	-	-	-	-	-	-	-	-
108	Nerolidol 1	-	-	-	-	-	-	-	0.49±0.02	-	-	-	-	-	-	-	-	-	-	-
109	*cis*-acetate-2-methylene-3-(1-methylethenyl)-Cyclohexanol	-	-	-	-	-	-	-	-	-	0.38±0.06	-	-	-	-	-	-	-	-	-
110	methyl ether-Nerol	-	-	-	-	-	-	-	-	-	-	-	-	-	-	-	0.44±0.02	-	-	-
111	(S)-(+)-1,2-Propanediol	-	-	-	1.02±0.11	-	-	-	-	-	-	-	-	-	-	-	-	-	-	-

Table S2. (continued).

No.	Compound	Concentration (μg/g)																		
		WZM	SMQ	XQG	NFM	GC	XJ	XN	ZM	CX	HY	DD	PS	LZ	BT	JP	XC	LF	YF	NHE
112	α,2,6,6-Tetramethyl-1-cyclohexene-1-methanol	-	-	-	-	-	-	-	-	-	-	-	-	-	-	-	-	1.97±0.03	2.08±0.17	-
113	(+/−)-1-amino-2-Propanol	-	-	-	-	-	-	-	-	-	-	0.34±0.01	-	-	-	-	-	-	-	-
114	*cis*-Linaloloxide	-	-	-	-	-	-	-	-	-	-	2.52±0.24	-	-	-	-	-	-	-	-
	Aldehydes																			
115	Dodecanal	-	-	-	-	-	-	-	-	-	-	-	-	0.70±0.06	-	-	-	0.60±0.03	-	-
116	Benzeneacetaldehyde	-	-	-	-	-	4.07±0.21	-	-	-	-	-	-	-	-	-	-	-	-	-
117	1,3-Dioxan-5-ol	0.06±0.0001	-	-	-	-	-	-	-	-	-	-	-	-	-	-	-	-	-	-
118	(E)-3,7-dimethyl-2,6-Octadienal	-	-	-	-	-	-	-	2.40±0.29	-	-	-	2.70±0.14	-	-	-	-	6.36±0.68	7.42±0.74	-
119	Nonanal	-	-	-	10.16±1.02	-	-	-	-	-	-	-	-	-	-	-	-	-	-	-
120	Octadecanal	-	-	-	-	-	-	-	-	0.74±0.06	-	-	-	-	-	-	-	-	-	-
121	Citronellal	-	0.95±0.04	-	-	-	-	-	2.25±0.37	-	-	-	-	-	-	-	-	-	-	-
122	Citral	-	-	-	-	-	-	-	-	-	-	-	-	2.09±0.31	-	-	-	-	-	-

123	Undecanal	-	-	-	-	-	-	-	-	1.13±0..16	0.51±0.02	-	0.39±0.04	-	-	-	-	-	1.74±0.07	1.81±.0.15
124	Octanal	-	-	3.64±0.36	15.19±2.18	-	-	-	4.74±0.32	-	-	-	-	-	-	-	-	-	-	-
125	(Z)-2-Decenal	-	-	-	-	-	-	-	-	-	-	-	0.81±0.01	-	-	-	-	-	-	-
126	(Z)-2-Butenal	12.01±0.84	7.17±1.04	-	-	12.73±1.09	8.69±0.18	-	-	-	6.67±0.27	27.94±1.24	-	-	29.60±0.37	-	-	18.68±2.04	24.31±1.75	-
127	Decanal	6.26±0.47	7.26±0.35	18.26±1.27	27.29±3.27	18.32±1.68	3.31±0.34	10.46±2.04	3.45±0.36	6.61±0.37	5.49±0.18	20.87±2.17	4.34±0.37	7.24±0.84	13.12±1.28	5.68±0.77	7.01±0.7	7.09±0.37	19.91±2.14	13.04±1.21
128	p-Menth-1-en-9-al	-	-	9.60±2.04	-	-	-	-	-	-	-	-	-	-	-	-	-	-	-	-
129	(S)-(−)-Perillaldehyde	-	6.98±0.75	17.35±2.077	-	-	-	-	-	-	-	-	-	-	-	-	-	-	-	-
130	(E)-2-Decenal	-	-	2.19±0.14	-	-	-	-	-	-	-	-	-	-	-	-	-	-	-	-
131	*L*-Perillaldehyde	4.07±0.35	-	-	16.50±1.24	14.94±2.15	5.29±1.05	9.87±2.14	6.16±1.42	4.94±0.75	2.28±0.27	17.68±0.87	2.81±0.11	4.13±0.85	17.41±0.36	4.85±0.38	5.17±0.25	3.76±0.37	11.28±1.08	9.54±0.24
132	2,6,9,11-Dodecatetraenal, 2,6,10-trimethyl-	-	0.07±0.0001	1.54±0.31	-	-	-	2.24±0.14	1.04±0.05	0.62±0.07	0.69±0.16	-	1.17±0.17	0.95±0.08	-	-	-	1.76±0.14	2.12±0.37	1.94±0.45
133	α-Sinensal	-	-	11.00±1.24	-	-	-	0.60±0.01	0.71±0.02	-	-	-	-	-	-	-	-	-	-	-

Table S2. (continued).

No.	Compound	Concentration (μg/g)																		
		WZM	SMQ	XQG	NFM	GC	XJ	XN	ZM	CX	HY	DD	PS	LZ	BT	JP	XC	LF	YF	NHE
134	Tridecanal	-	-	1.89±0.24	-	9.88±0.24	-	-	-	-	-	-	-	-	-	-	-	-	-	-
135	2-methyl-3-methylene-Cyclopentanecarboxaldehyde	-	16.97±2.31	-	-	-	-	-	-	-	-	-	-	-	-	-	-	-	-	-
136	4-Cyclohexylidenebutyraldehyde	-	-	124.62±4.21	-	-	-	-	-	-	-	-	-	-	-	-	-	-	-	-
137	Benzaldehyde	-	-	-	-	-	-	-	-	-	-	0.39±0.02	-	-	-	-	-	-	-	-
138	(+)-Citronellal	-	-	-	2.73±0.27	-	-	-	-	-	-	-	-	-	-	-	-	-	-	-
139	dimethoxy-Methane	-	-	-	-	-	-	-	-	-	-	-	-	-	-	-	-	0.97±0.04	1.25±0.27	-
140	3,5-Dimethylcyclohex-1-ene-4-carboxaldehyde	-	-	-	-	-	-	-	-	-	-	-	-	-	-	-	-	0.57±0.02	0.73±0.03	-
	Ketones																			
141	(+)-Dihydrocarvone	5.92±1.03	-	7.44±0.68	-	-	-	-	-	2.35±0.28	-	-	-	-	-	-	5.50±0.45	-	-	-
142	Hydroxyacetone	-	-	-	-	-	-	-	0.28±0.02	-	-	-	-	-	-	-	-	-	-	-
143	2-(1-methylpropyl)-Cyclopentanone	-	-	-	18.76±2.61	-	-	-	-	9.62±1.24	-	-	-	20.03±3.17	-	-	-	-	-	-

144	2-Hydroxy-5-methyl acetophenone	1.31±0.21	-	-	0.44±0.01	-	-	-	-	-	-	-	-	-	-	-	-	-	-	-
145	4-Isopropenylcyclohexanone	-	-	-	-	-	-	-	-	2.79±0.23	-	-	-	3.75±0.27	-	-	-	-	-	-
146	Hydroxyacetone	-	-	-	-	-	-	-	-	-	-	-	-	-	-	-	-	12.37±2.17	-	-
147	[1,1'-Bicyclopentyl]-2-one	-	-	-	0.75±0.27	-	0.57±0.07	0.54±0.04	-	-	-	-	-	0.45±0.06	-	0.78±0.18	-	-	-	1.28±0.24
148	*trans*-Dihydrocarvone	-	2.90±0.37	-	-	28.28±2.31	18.15±2.08	3.55±0.37	-	-	0.90±0.14	-	-	2.98±0.19	18.44±2.31	1.79±0.57	-	-	3.71±0.93	-
149	3-Methoxyacetophenone	-	-	-	-	-	-	0.54±0.18	-	-	-	-	-	-	-	2.23±0.47	-	-	-	-
150	3,5-Octadien-2-one	-	1.25±0.27	-	-	-	-	-	0.74±0.27	-	0.47±0.03	-	-	-	-	2.18±0.57	5.67±1.06	-	-	-
151	*D*(+)-Carvone	23.01±2.37	15.57±0.37	47.23±2.17	25.31±2.57	98.32±3.28	33.30±3.21	24.08±1.27	14.52±2.34	23.17±3.23	13.12±2.18	61.32±3.58	18.72±1.27	24.48±2.16	92.50±0.57	22.81±3.21	33.44±2.31	-	31.24±2.18	29.80±3.57
152	Piperiton	-	-	1.16±0.07	0.68±0.03	-	-	0.79±0.04	0.85±0.17	-	-	-	-	0.50±0.01	-	1.69±0.07	-	-	1.29±0.91	-
153	*β*-Lonone	-	-	-	-	-	-	-	1.28±0.27	-	-	-	1.63±0.34	-	3.53±0.57	-	1.42±0.09	-	3.20±0.37	-
154	6,10,14-trimethyl-2-Pentadecanone	-	-	-	-	-	-	0.63±0.04	-	-	-	-	-	0.51±0.18	-	-	-	0.67±0.14	0.89±0.18	1.23±0.67
155	*β*-Ionone	-	-	3.92±0.38	4.01±0.87	6.30±0.68	-	2.79±1.03	-	2.00±0.37	-	-	-	1.62±0.27	-	-	-	1.21±0.18	-	3.74±1.08
156	Jasmolin I	-	-	-	-	9.34±1.37	7.69±1.05	1.58±0.06	-	2.00±0.24	5.14±0.68	17.54±2.27	-	0.86±0.29	-	2.42±0.37	-	-	-	3.05±0.37

Table S2. (continued).

No.	Compound	Concentration (μg/g)																		
		WZM	SMQ	XQG	NFM	GC	XJ	XN	ZM	CX	HY	DD	PS	LZ	BT	JP	XC	LF	YF	NHE
157	4-Hydroxy-3-methylacetophenone	-	-	-	-	-	-	-	-	-	-	5.61±1.21	-	-	-	-	-	-	-	-
158	*α*-Ionone	-	0.84±0.16	-	0.49±0.02	-	-	-	-	-	-	-	0.25±0.02	-	-	-	-	-	-	-
159	4-Methylacetophenone	-	-	-	-	14.40±2.09	-	-	-	-	-	-	-	-	-	-	-	-	-	-
160	1-(3-methylphenyl)-Ethanone	-	-	0.99±0.27	-	-	-	-	-	-	-	-	-	-	-	-	-	-	-	-
161	3-Methyl-2-cyclohexen-1-one	-	-	15.45±2.19	-	-	-	-	-	-	-	-	-	-	-	-	-	-	-	-
162	(E,E)-3,5-Octadien-2-one	-	-	-	-	-	-	-	-	2.95±0.98	-	-	-	-	-	2.58±0.19	-	-	-	-
163	(4S)-1,8-p-Menthadiene-3-one	-	-	-	4.84±0.24	-	-	-	-	-	-	4.29±0.37	-	-	3.67±0.21	-	-	-	-	-
164	4-Hydroxy-2-methylacetophenone	-	-	-	-	-	-	-	-	-	-	-	-	-	-	-	-	-	-	2.59±0.64
165	6-Methylhept-5-en-one	2.18±0.28	-	-	-	-	-	0.77±0.06	-	-	-	-	0.07±0.002	0.15±0.03	-	-	-	-	1.43±0.28	-
166	5-[1,2,4]Triazol-1-yl-pyrrolidin-2-one	-	-	-	-	-	-	14.55±2.06	-	-	-	-	10.58±1.35	-	-	-	-	-	-	-
167	1,3-Bis(cyclopentyl)-1-cyclopentanone	1.81±0.06	-	-	-	-	-	-	-	1.39±0.21	-	-	0.69±0.03	-	-	-	-	-	-	-
168	3,5-dimethyl-2-Cyclohexen-1-one	-	-	-	-	-	-	-	-	1.62±0.16	-	-	-	-	-	-	-	-	-	-

169	2-(2-propenyl)-Cyclohexanone	-	-	-	-	-	-	-	-	-	2.65± 0.31	-	-	-	-	-	-	1.98± 0.12	1.68± 0.16	-
170	3-Hydroxy-2,3-dihydromaltol	-	-	-	-	-	-	-	1.72± 0.18	-	-	-	-	-	-	-	-	-	-	-
171	4-[2-isopropyl-5-methyl-5-(2-methyl-5-oxocyclopentyl)cyclopentenyl]-2-Butanone	-	-	-	2.35± 0.78	-	-	-	-	-	-	-	-	-	-	-	-	-	-	-
172	2-(2-butynyl)-Cyclohexanone	-	-	-	1.68±. 0.15	-	-	-	-	-	-	-	-	-	-	-	-	-	-	-
173	7-methyl-5-Octen-4-one	-	-	-	2.41± 0.18	-	-	-	-	-	-	-	-	-	-	-	-	-	-	-
174	3-Ethoxyphenylacetone hydroxyoxime	-	-	-	-	-	-	-	-	-	-	-	-	-	-	-	-	0.69± 0.06	0.85± 0.18	-
	Hydrocarbons																			
175	1r-.α.-Pinene	-	-	-	-	-	-	-	7.37± 0.37	-	-	-	1.42± 0.18	-	-	7.30± 0.67	6.30± 0.18	-	-	0.86± 0.08
176	pinene	-	-	-	-	-	-	-	-	2.38± 0.48	-	-	-	-	-	-	-	-	-	-
177	1s-.α.-Pinene	-	-	-	-	-	-	-	-	-	-	1.54± 0.08	-	1.40± 0.04	-	-	-	-	-	-
178	Sabinene	-	-	8.98± 1.04	-	-	7.61± 0.28	7.32± 1.03	-	-	-	-	2.43± 0.08	3.28± 0.19	1.16± 0.08	-	7.57± 1.28	-	-	11.87 ±1.28
179	(1S)-(−)-β-Pinene	-	7.34± 0.27	-	18.69 ±2.18	2.12± 0.57	-	-	-	-	-	2.36± 0.07	-	-	-	-	-	-	-	-
180	(+)-4-Carene	-	-	-	-	-	-	-	3.34	-	-	-	-	-	-	-	-	-	-	-

Table S2. (continued).

No.	Compound	Concentration (μg /g)																		
		**WZM**	**SMQ**	**XQG**	**NFM**	**GC**	**XJ**	**XN**	**ZM**	**CX**	**HY**	**DD**	**PS**	**LZ**	**BT**	**JP**	**XC**	**LF**	**YF**	**NHE**
181	*β*-Terpinene	-	-	-	0.72±0.02	-	-	-	-	-	-	-	-	-	-	-	-	0.84±0.02	0.69±0.05	-
182	*α*-Pinene	3.05±1.02	8.79±1.07	11.36±2.03	7.80±1.27	3.94±0.36	3.32±1.03	3.19±0.75	12.83±1.45	5.80±0.57	3.59±0.37	3.32±0.74	0.87±0.18	0.98±0.05	2.23±0.43	1.23±0.36	2.36±0.27	1.36±0.45	2.77±0.14	0.67±0.34
183	*β*-Pinene	8.92±0.87	-	-	-	-	-	-	-	4.95±0.96	-	-	-	-	-	7.78±0.37	-	-	-	-
184	1,3,8-p-Menthatriene	3.79±0.27	-	50.46±2.37	4.13±1.33	47.41±3.87	31.39±2.1	-	-	6.67±0.18	1.92±0.24	-	1.93±0.27	24.05±3.57	33.03±3.14	-	4.50±0.34	3.29±0.21	4.44±0.37	38.29±1.27
185	2-Isopropenyl-5-methylhex-4-enal	-	-	-	-	-	-	-	-	-	-	-	-	-	-	-	21.75±2.17	-	-	-
186	2,5,5-trimethyl-1,3,6-Heptatriene	-	-	-	-	-	-	-	-	-	-	-	-	-	-	1.31±0.32	-	-	-	-
187	*β*-Myrcene	29.22±3.21	22.88±3.27	17.40±2.09	35.59±1.98	26.17±3.17	19.63±1.98	17.59±2.18	17.29±2.33	24.22±6.28	17.32±4.29	55.50±6.32	11.63±327	12.96±2.04	32.03±4.16	25.65±2.09	29.89±3.27	13.90±2.31	22.93±2.32	30.50±2.34
188	*D*-Limonene	803.52±35.26	754.91±24.16	779.25±35.98	901.34±45.24	1594.93±24.75	843.03±35.26	610.88±17.24	509.67±14.26	674.52±32.06	439.80±10.24	1330.21±35.09	369.77±17.65	492.73±20.37	2037.63±38.97	948.59±34.87	974.59±45.09	410.53±17.24	855.16±35.29	1069.29±26.56
189	*β*-Ocimene	3.68±0.39	6.55±1.21	4.19±0.47	5.69±0.34	7.26±1.01	4.31±0.18	8.16±0.73	9.64±1.34	6.67±0.39	3.19±0.24	14.25±1.28	11.03±1.77	2.76±0.22	3.61±0.38	7.09±0.27	1.36±0.06	3.64±0.33	4.26±0.19	1.11±0.02
190	2,5,5-trimethyl-1,5-Cyclooctadiene	-	-	-	-	-	-	-	-	-	2.33±0.27	-	-	-	-	-	-	-	-	-
191	(Z)-*β*-Ocimene	5.57±0.68	3.17±0.38	8.94±1.06	-	7.83±2.19	-	3.39±1.06	2.90±0.37	-	3.09±0.57	16.88±2.01	1.99±0.24	-	6.34±0.39	-	4.16±0.37	4.01±0.18	3.16±0.24	1.20±0.05

192	γ-Terpinene	62.34±5.68	109.26±7.68	34.02±2.67	75.80±6.32	68.25±5.27	21.47±2.07	44.57±2.37	60.61±3.24	64.83±3.57	35.52±2.18	20.35±3.27	12.41±2.18	17.15±1.08	37.56±2.27	96.58±3.57	25.09±3.74	8.18±1.09	28.18±3.27	27.76±2.19
193	Terpinolene	-	6.70±0.67	-	-	-	-	-	-	0.68±0.06	-	-	-	-	-	-	-	-	-	-
194	Cosmene	-	-	1.82±0.37	-	9.45±1.32	24.80±2.37	-	-	2.76±0.37	13.00±3.24	-	-	-	27.55±3.24	-	-	-	-	9.85±1.98
195	2-Methyl-3-trans-propenylpyrazine	-	-	-	-	-	-	-	-	-	-	-	-	-	6.12±1.32	-	-	-	-	2.77±0.35
196	4-Acetyl-1-methyl-1-cyclohexene	-	-	-	-	0.69±0.21	1.77±0.68	-	-	-	-	-	0.03±0.0002	-	-	-	0.04±0.0001	-	-	-
197	3-Carene	3.18±1.03	-	-	-	-	-	-	-	-	-	-	-	-	-	-	-	-	-	-
198	2-methyl-6-methylene-1,7-Octadien-3-one	4.53±0.21	-	-	-	-	-	-	-	-	-	-	1.67±0.35	-	-	-	2.63±0.36	-	-	-
199	1,4-Pentadiene	-	-	-	-	-	-	-	-	-	-	-	-	-	-	-	3.10±0.37	-	-	-
200	1,Z-5,E-7-Dodecatriene	-	3.09±0.31	-	-	-	-	-	-	-	-	-	-	-	-	-	-	-	-	-
201	α-Humulene	-	-	-	-	-	-	-	-	-	-	21.51±2.35	-	-	-	-	-	1.49±0.02	-	-
202	*cis*-β-Farnesene	-	-	-	-	-	-	-	-	-	-	27.38±4.32	-	-	-	-	-	24.68±2.35	-	-
203	Cyclohexene, 2-ethenyl-1,3,3-trimethyl-	-	-	-	-	-	-	4.17±1.27	-	-	-	9.37±2.03	-	-	-	-	-	7.65±0.67	-	-

Table S2. (continued).

No.	Compound	Concentration (μg/g)																		
		**WZM**	**SMQ**	**XQG**	**NFM**	**GC**	**XJ**	**XN**	**ZM**	**CX**	**HY**	**DD**	**PS**	**LZ**	**BT**	**JP**	**XC**	**LF**	**YF**	**NHE**
204	Bicyclo[4.1.0]hept-2-ene, 3,7,7-trimethyl-	-	-	13.45±3.27	5.28±2.03	-	-	-	-	-	1.49±0.27	-	-	-	-	10.84±2.03	-	-	-	-
205	Longifolene-(V4)	-	-	0.60±0.01	-	-	-	-	-	-	-	-	-	-	-	-	-	-	-	-
206	*α*-Cubebene	2.85±0.32	5.71±1.23	2.14±0.32	3.26±1.02	15.85±3.65	6.59±1.02	1.74±0.36	1.20±0.03	5.66±0.57	2.43±0.21	17.01±3.21	1.86±0.23	1.97±0.21	10.10±1.94	5.43±0.37	1.81±0.26	2.16±0.07	2.58±0.35	5.60±1.03
207	2,6-dimethyl-2,6-Octadiene	5.16±0.24	2.74±0.16	3.92±0.35	5.89±1.06	14.92±1.62	2.35±0.21	3.62±1.23	2.84±0.36	4.18±1.06	2.81±0.67	13.85±2.06	2.91±0.28	4.53±1.03	10.64±2.06	3.73±0.67	6.54±1.06	2.43±0.24	6.24±0.26	8.19±2.14
208	1,6-dimethyl-1,5-Cyclooctadiene	-	-	0.75±0.01	-	-	-	-	-	-	0.65±0.06	-	-	-	-	0.51±0.02	-	-	-	-
209	Copaene	3.42±0.343	4.95±1.03	1.91±0.06	4.30±0.37	8.60±1.37	0.70±0.02	2.48±1.06	2.35±0.16	4.11±0.68	3.07±1.26	12.23±1.26	2.50±0.24	2.41±0.35	4.37±0.34	5.71±0.39	1.87±0.34	2.68±0.16	3.64±1.03	5.81±0.37
210	(−)-*β*-Elemene	25.96±5.64	63.45±6.98	24.22±2.15	12.85±2.36	50.17±6.37	29.91±6.24	8.54±1.17	7.35±1.26	13.85±1.35	13.26±2.06	64.71±6.49	8.69±0.67	9.15±1.26	16.65±2.09	27.51±2.18	6.58±0.36	5.22±0.29	10.38±1.26	19.26±1.32
211	*β*-Caryophyllene	7.08±0.79	12.99±2.06	8.93±0.68	8.48±0.37	15.01±1.26	2.79±0.27	5.04±1.06	4.38±0.36	11.45±2.18	-	43.72±5.18	7.51±0.38	4.19±0.37	14.02±1.37	23.69±2.19	3.26±1.02	2.69±0.35	6.45±1.36	9.45±0.26
212	*α*-Terpinene	-	-	-	-	-	-	-	-	-	0.27±0.03	-	-	-	-	0.59±0.07	-	-	-	-
213	(−)-Germacrene D	3.63±0.64	16.52±1.37	-	8.77±1.24	8.88±0.36	6.79±1.02	-	2.54±0.12	8.59±1.36	20.63±2.47	35.59±3.58	1.01±0.05	4.13±0.34	5.01±1.02	8.43	0.88±0.07	0.93±0.12	5.32±1.07	4.76±0.25
214	*γ*-Elemene	2.55±0.54	8.32±1.21	4.48±1.21	4.35±0.35	10.83±1.57	8.87±1.36	2.37±1.27	1.37±0.34	5.78±0.15	9.79±1.24	32.98±3.54	2.36±0.12	1.55±0.07	3.80±0.58	12.80±1.27	0.66±0.05	1.09±0.08	1.75±0.37	4.42±0.57
215	1,1,7-Trimethyl-4-methylenedecahydro-1H-cyclopropa[e]azulene	-	1.20±0.12	0.77±0.04	-	-	-	-	-	0.96±0.12	11.63±1.34	9.59±1.25	-	-	-	3.28±0.15	-	-	1.03±0.57	0.84±0.05

216	Z,Z,Z-1,5,9,9-tetramethyl-1,4,7,-Cycloundecatriene	5.30±0.34	13.57±2.04	4.61±0.45	5.36±0.18	14.79±0.34	9.11±0.24	2.90±0.48	1.83±0.08	5.03±1.02	4.70±1.04	-	2.72±1.04	2.37±0.64	7.03±1.45	8.64±1.02	1.79±0.07	3.29±0.34	4.12±0.15	6.57±0.67
217	*trans*-*β*-Farnesene	5.72±0.18	6.15±0.27	5.44±0.18	6.80±0.34	-	2.70±0.08	4.98±0.37	8.09±0.27	12.47±2.15	5.69±0.32	-	14.91±1.54	3.35±0.47	14.73±2.15	35.04±2.38	3.37±0.34	3.26±0.47	6.20±1.02	7.57±0.89
218	(+)-Epi-bicyclosesquiphellandrene	-	-	-	-	-	-	-	-	0.99±0.18	0.81±0.04	-	-	-	-	-	-	0.76±0.18	-	0.55±0.06
219	Isoledene	-	0.96±0.06	-	-	-	-	-	-	-	0.51±0.02	-	-	-	-	-	-	-	-	-
220	(+)-*β*-Selinene	4.88±0.31	-	2.39±0.16	-	-	-	-	-	-	-	-	-	-	-	-	-	-	-	-
221	*α*-Farnesene	-	27.20±3.26	-	40.48±3.64	31.21±2.34	27.65±3.14	-	5.94±0.46	9.97±0.35	-	24.94±1.21	-	8.67±1.24	-	-	-	14.38±2.01	17.04±1.05	20.50±3.15
222	2-Isopropyl-5-methyl-9-methylene[4.4.0]dec-1-ene	-	-	-	-	-	-	-	-	-	0.33±0.03	2.77±0.54	-	-	-	-	-	-	-	0.52±0.02
223	(+)-Cycloisosativene	-	1.95±0.21	-	-	-	-	-	-	-	-	-	-	-	-	-	-	-	-	-
224	(−)-Humulene epoxide II	1.94±0.32	-	-	-	-	-	0.64±0.02	-	-	-	2.92±0.32	-	-	-	-	-	-	-	-
225	1,7,7-Trimethyl-2-vinylbicyclo[2.2.1]hept-2-ene	5.44±0.65	-	-	6.17±1.03	-	-	-	-	-	-	-	-	-	-	-	-	-	-	-
226	(+)-Valencene	-	-	-	-	2.81±0.26	1.25±0.31	-	-	-	-	-	0.32±0.08	-	-	1.42±0.65	0.59±0.03	-	-	-
227	*γ*-Maaliene	1.10±0.21	-	-	-	5.60±1.32	2.36±0.6	2.11±0.2	-	2.30±0.31	1.61±0.24	12.53±2.3	-	-	-	-	0.94±0.32	0.77±0.31	0.36±0.01	-
228	endo-8-hydroxy-Cycloisolongifolene	-	1.25±0.24	-	-	-	-	-	-	-	0.61±0.06	4.12±0.32	-	-	-	-	-	-	-	-

Table S2. (continued).

No.	Compound	Concentration (μg /g)																		
		WZM	SMQ	XQG	NFM	GC	XJ	XN	ZM	CX	HY	DD	PS	LZ	BT	JP	XC	LF	YF	NHE
229	7R,8R-8-Hydroxy-4-isopropylidene-7-methylbicyclo[5.3.1]undec-1-ene	-	-	1.73± 0.36	-	-	-	-	-	-	-	-	-	-	-	-	-	-	-	-
230	*cis*-Z-*α*-Bisabolene epoxide	2.54± 0.36	-	1.74± 0.41	-	-	-	-	-	-	-	-	-	-	-	1.02± 0.32	-	-	-	0.95± 0.03
231	(+)-(1R,4R)-Isolimonene	-	0.54± 0.08	-	-	-	-	-	-	-	-	-	-	-	-	-	-	-	-	-
232	Eremophilene (7CI)	-	-	-	1.26± 0.21	-	-	-	-	-	1.59± 0.41	-	-	-	-	-	-	-	-	-
233	(3Z,6E)-*α*-Farnesene	-	-	-	-	-	-	-	-	-	-	-	0.28± 0.02	-	-	-	-	-	-	-
234	4,11,11-trimethyl-8-methylene-Bicyclo[7.2.0]undec-4-ene	-	-	1.60± 0.12	-	-	-	-	-	-	-	-	-	-	-	-	-	-	-	-
235	*β*-Panasinsene	-	-	3.89± 0.16	-	-	-	-	-	-	-	-	-	-	-	-	-	-	-	-
236	7-endo-ethenyl-Bicyclo[4.2.0]oct-1-ene	-	1.74± 0.32	-	-	-	-	-	-	-	-	-	-	-	-	-	-	-	-	-
237	Germacrene B	-	-	-	-	-	-	-	-	-	-	-	2.56± 0.31	-	-	-	-	-	-	-
238	*β*-Thujene	-	-	-	-	-	-	-	-	-	-	-	-	-	-	-	-	4.68± 1.21	6.41± 1.36	-
239	*α*-Muurolene	-	-	-	-	-	-	-	-	0.50± 0.01	-	-	-	-	-	-	-	-	-	-

240	(−)-*β*-Chamigrene	-	-	-	-	-	-	-	-	-	-	-	-	-	-	2.28±0.19	-	-	-	-
241	(+)-Ledene	-	-	-	-	3.13±0.18	1.69±0.35	-	-	-	-	-	-	-	-	0.99±0.01	-	-	-	-
242	3,9-Epoxy-p-mentha-1,8(10)-diene	4.64	-	-	-	-	-	-	-	3.02±0.76	-	-	-	-	-	-	-	-	-	-
243	(+)-1(10)-Aristolene	-	-	-	-	-	-	-	-	2.35±0.84	3.32±1.02	-	-	-	-	-	-	-	-	-
244	7,11-Dimethyl-3-methylene-1,6,10-dodecatriene	-	-	-	-	10.44±2.1	-	-	-	-	-	-	-	-	-	-	-	-	-	-
245	3-methyl-4-methylene-Bicyclo[3.2.1]oct-2-ene	-	-	-	-	9.62±0.35	-	-	-	-	-	-	-	1.55±0.31	-	4.33±0.87	-	-	-	-
246	*β*-Phellandrene	-	-	-	-	-	-	-	10.88±2.07	-	6.05±1.02	-	-	-	-	-	-	-	-	-
247	4-Methyl-1,4-heptadiene	-	-	-	-	-	-	-	-	-	1.35±0.05	-	2.86±0.2	-	-	-	1.92±0.21	2.11±0.17	1.68±0.15	-
248	10s,11s-Himachala-3(12),4-diene	-	-	-	-	-	-	-	-	-	-	3.69±0.32	0.53±0.01	-	-	-	-	-	-	-
249	Ledene oxide-(II)	-	21.69±2.13	-	-	-	-	-	-	-	2.69±0.21	-	-	-	-	-	-	-	-	-
250	R(−)3,7-Dimethyl-1,6-octadiene	1.90±0.02	-	-	-	-	-	-	-	-	-	-	-	-	-	-	-	-	-	-
251	zingiberene	-	-	-	-	-	-	-	-	-	-	-	-	-	-	1.41±0.03	-	-	-	-
252	(E,Z)-2,6-Dimethylocta-2,4,6-triene	-	-	-	-	2.69±0.23	6.55±1.31	-	-	0.94±0.06	-	-	-	-	-	-	-	-	-	-
253	3-ethenyl-1,2-dimethyl-1,4-Cyclohexadiene	-	-	-	-	-	-	3.98±0.57	-	-	-	-	-	-	-	-	-	-	-	-

Table S2. (continued).

No.	Compound	Concentration (μg/g)																		
		WZM	SMQ	XQG	NFM	GC	XJ	XN	ZM	CX	HY	DD	PS	LZ	BT	JP	XC	LF	YF	NHE
254	*D*-Longifolene	0.50±0.01	-	-	-	-	-	-	-	-	-	-	-	-	-	-	-	-	-	-
255	(E,E)-1,3,5-Undecatriene	-	-	0.37±0.02	-	-	-	-	-	-	-	-	-	-	-	-	-	-	-	-
256	(+)-*α*-Elemene	-	-	-	-	-	-	-	-	-	-	-	-	-	-	0.64±0.12	-	-	-	0.93±0.12
257	8,9-dehydro-Cycloisolongifolene	-	-	-	-	-	-	-	-	-	-	-	0.23±0.02	-	-	1.02±0.08	-	-	-	-
258	*cis*-(−)-2,4a,5,6,9a-Hexahydro-3,5,5,9-tetramethyl(1H)benzocycloheptene	-	-	-	-	-	-	-	-	-	-	-	-	-	-	4.05±1.14	-	-	-	-
259	Cedrene	-	-	-	-	-	-	-	-	-	-	-	-	-	-	1.44±0.35	-	-	-	-
260	3-Thujene	-	-	-	-	-	0.95±0.08	-	-	-	-	-	-	-	-	-	-	-	-	-
261	Limonene-1,2-epoxide	-	-	-	-	-	0.11±0.0002	-	-	-	-	-	-	-	-	-	-	-	-	-
262	4,7-dimethyl-Decan-2,4,8-triene	-	-	-	-	-	1.36±0.23	-	-	-	-	-	-	-	-	-	1.91±0.35	-	-	2.22±0.78
263	(Z)-1,4,9-Decatriene	-	-	-	-	-	-	-	-	-	-	-	2.48±0.64	-	-	-	-	-	-	-
264	5,5-dimethyl-1,3-Heptadiene	4.08±1.02	-	-	-	-	-	-	-	-	-	-	-	-	-	-	-	-	-	-

265	1-chloro-2-methyl-2-Butene	11.51±2.01	-	-	-	-	-	-	-	-	-	-	-	-	-	-	-	-	-	-
266	2,4,4-Trimethyl-3-hydroxymethyl-5a-(3-methyl-but-2-enyl)-cyclohexene	1.09±0.03	-	-	-	-	-	-	-	-	-	-	-	-	-	-	-	-	-	-
267	7,8-dehydro-8a-hydroxy-Isolongifolene	0.63±0.02	-	-	-	-	-	-	-	-	-	-	-	-	-	-	-	-	-	-
268	1-methyl-5-(1-methylethenyl)-Cyclohexene	-	-	-	-	-	-	2.73±0.46	1.49±0.13	-	1.92±0.08	-	-	1.84±0.35	-	-	-	2.78±0.15	3.16±0.75	-
269	Bicyclo[6.1.0]non-1-ene	-	-	-	-	-	-	-	2.57±0.21	-	-	-	-	-	-	-	-	-	-	-
270	1,1-dichloro-3,4-dimethyl-Germacyclopent-3-ene	-	-	-	-	-	-	-	-	-	0.77±0.03	-	-	-	-	-	-	-	-	-
271	Ledene oxide (I)	-	-	-	-	-	-	-	-	-	0.43±0.04	-	-	-	-	-	-	-	-	-
272	Cyclohexene, 1-methyl-5-(1-methylethenyl)-, (R)-	-	-	-	3.02±0.67	-	-	-	-	-	-	-	-	-	-	-	-	-	-	-
273	2-Octene, 2-methyl-6-methylene-	-	-	-	4.44±0.45	-	-	-	-	-	-	-	-	-	-	-	-	-	-	-
274	Bicyclopentyl-1,1'-diene	-	-	-	54.49±2.36	-	-	-	-	-	-	-	-	-	-	-	-	-	-	-
275	(E,E)-7,11,15-Trimethyl-3-methylene-hexadeca-1,6,10,14-tetraene	-	-	-	-	-	-	-	-	-	-	-	-	-	-	-	-	2.97±0.14	4.36±0.36	-

Table S2. (continued).

No.	Compound	Concentration (μg /g)																		
		WZM	SMQ	XQG	NFM	GC	XJ	XN	ZM	CX	HY	DD	PS	LZ	BT	JP	XC	LF	YF	NHE
276	1-(1,4-dimethyl-3-cyclohexen-1-yl)-Ethanone	-	-	-	-	-	-	-	-	-	-	-	-	-	-	-	-	1.98±0.32	1.68±0.12	-
277	Caryophyllene (I1)	-	-	-	-	-	-	-	-	-	-	8.05±1.36	-	-	-	-	-	-	-	-
278	Tricyclo[2.2.2.0(1,4)]octane	-	-	-	0.85±0.21	-	-	-	-	-	-	-	-	-	-	-	-	-	-	-
279	Alloaromadendrene oxide-(1)	-	-	-	-	-	-	-	-	-	-	-	-	-	-	1.34±	-	-	-	-
280	Ethylene oxide	-	0.43±	-	-	-	-	-	-	-	-	-	-	-	-	-	-	-	-	-
281	Tricyclo[4.4.1.0(1,6)]undecane	-	0.59±0.06	-	43.46±4.29	-	-	-	-	-	-	157.73±6.37	-	-	-	-	54.77±7.21	-	-	-
282	2,3-Epoxypinane	55.27±6.25	-	-	-	219.69±15.21	125.32±7.64	-	-	-	-	-	-	-	202.28±16.24	-	-	-	-	-
283	7-(1-methylethylidene)-Bicyclo[4.1.0]heptane	-	-	-	-	3.70±0.23	1.02±0.12	-	-	-	0.30±0.01	-	-	-	-	-	-	-	-	-
284	8-(1-methylethylidene)-Bicyclo[5.1.0]octane	-	-	-	-	-	-	-	-	0.45±0.08	-	-	-	-	-	-	-	-	-	-
285	Isoaromadendrene epoxide	0.70±0.02	3.21±0.23	2.28±0.64	-	2.53±0.12	1.36±0.11	-	-	-	-	-	0.77±0.31	-	-	0.91±0.08	-	-	-	0.91±0.02

286	Cyclodecane	-	-	-	6.12±0.21	-	-	-	-	-	-	4.19±0.65	-	-	-	-	-	-	-	1.59±0.31
287	9-(1-methylethylidene)-Bicyclo[6.1.0]nonane	1.89±0.35	-	-	-	-	-	-	-	-	-	-	-	-	-	-	-	-	-	-
288	Cyclooctane	-	-	-	-	-	-	-	-	-	-	10.17±1.36	-	-	-	6.39±±0.65	-	-	-	-
289	*cis*-3-cyclopropyl,-7-hydroxymethyl-Bicyclo[4.1.0]heptane	-	-	19.28±2.67	-	14.18±1.32	2.64±0.01	-	-	-	-	-	-	-	-	-	-	-	-	-
290	1,5-dimethyl-2,6-bis(methylene)-Cyclooctane	-	-	-	-	9.30±1.23	10.36±1.24	-	-	-	-	-	-	-	-	-	-	-	-	-
291	7-Propylidene-bicyclo[4.1.0]heptane	-	-	-	-	-	-	-	-	-	-	-	-	-	-	4.81±0.32	-	-	-	-
292	2-chloro-2-nitro-Propane	-	-	-	-	35.14±3.64	40.74±1.68	-	-	-	-	-	-	-	-	-	-	-	-	-
293	1,1-dimethyl-2-(1-methyl-2-propenyl)-Cyclopropane	-	-	-	-	-	1.44±0.32	-	-	-	-	-	-	-	-	-	-	0.58±0.01	0.37±0.03	-
294	6-methylene-Spiro[4.5]decane	-	-	-	-	-	-	-	-	-	-	-	-	-	-	-	-	-	-	0.76±0.21
295	Tetradecane	-	-	-	-	-	-	-	-	-	-	-	0.12±0.0008	-	-	-	-	-	-	0.46±0.02
296	erythro-9,10-Dibromopentacosane	-	-	-	-	-	-	-	-	-	-	-	0.32±0.003	-	-	-	-	-	-	-
297	6-methylene-Methane	-	-	-	-	-	-	0.17±0.004	-	-	-	-	-	-	-	-	-	-	-	-
298	heptadecyl-Oxirane	-	-	-	-	-	-	-	1.55±0.32	-	-	-	-	-	-	-	-	-	-	-

Table S2. (continued).

No.	Compound	Concentration (μg /g)																		
		WZM	SMQ	XQG	NFM	GC	XJ	XN	ZM	CX	HY	DD	PS	LZ	BT	JP	XC	LF	YF	NHE
299	heptadecyl-2-Propanamine	-	-	-	-	-	-	-	-	-	0.05±0.0002	-	-	-	-	-	-	-	-	-
300	4-Methyl-1-(1-methylethyl)bicyclo[3.1.0]hexane didehydro deriv.	-	-	-	-	-	-	-	-	-	0.49±0.01	-	-	-	-	-	-	-	-	-
301	6,6-Dimethyl-3-methylenenorpinane	-	-	-	-	-	-	-	-	-	0.28±0.006	-	-	-	-	-	-	-	-	-
302	1-methyl-4-methylene-Cyclohexane	-	-	-	-	-	-	-	-	-	-	-	-	-	-	-	1.80±0.21	-	-	-
303	*D*-Camphene	-	-	-	4.91±0.64	-	-	-	-	-	-	-	-	-	-	-	-	-	-	-
304	diacetate1,1-Dodecanediol	-	-	-	2.39±0.21	-	-	-	-	-	-	-	-	-	-	-	-	-	-	-
305	Tetracyclo[3.3.1.1(1,8).0(2,4)]decane	-	-	-	-	-	-	-	-	-	-	7.75±0.97	-	-	-	-	-	-	-	-
	Benzene derivative																			
306	2,3,4,6-Tetramethylphenol	-	-	-	-	-	-	-	-	-	-	-	-	-	-	-	-	3.75±0.34	5.73±0.64	-
307	methoxy-phenyl-Oxime	-	59.83±3.24	15.66±1.32	22.19±2.74	3.59±1.04	8.45±1.35	24.81±4.01	16.50±0.38	5.17±0.24	-	-	10.92±2.06	0.10±0.001	-	18.48±2.31	18.61±2.19	26.40±2.64	29.19±2.31	-
308	6,7-dimethoxy-2-(4-methoxyphenyl)-4-propyl-4H-3,1-Benzoxazine	-	-	0.93±0.01	-	-	-	0.80±0.02	-	-	-	-	-	-	-	-	-	-	-	-

309	7-Chloro-2,3-dihydro-3-(4-N,N-dimethylaminobenzylidene)-5-phenyl-1H-1,4-benzodiazepin-2-one	-	-	-	1.13±0.02	-	-	1.08±0.32	-	0.19±0.001	-	-	-	-	-	-	-	-	-	-
310	2-Chloro-4-(4-methoxyphenyl)-6-(4-nitrophenyl)pyrimidine	-	0.75±0.01	-	-	-	-	-	-	-	-	-	-	-	-	-	0.76±0.02	-	-	-
311	3,5-Di-tert-butylcatechol	-	0.79±0.01	-	-	-	-	-	-	-	-	-	-	-	-	-	-	-	-	-
312	4-ethyl-2-methoxy-Phenol	-	-	-	-	11.65±1.03	9.48±1.32	-	-	-	-	-	-	-	-	-	-	-	-	-
313	3-Methyl-4-isopropylphenol	8.71±1.32	31.91±4.65	-	-	66.93±2.31	39.90±3.25	-	-	5.75±0.64	-	10.45±2.01	-	-	18.70±1.36	23.51±3.26	-	1.65±0.35	0.78±0.08	-
314	2-Methoxy-4-vinylphenol	-	-	2.23	-	193.95±7.36	65.61±3.64	-	3.07±0.32	2.45±0.36	-	-	1.48±0.23	-	113.78±9.34	-	-	-	-	-
315	9-Amino-7-mercapto-5,6,8,10-tetraaza-benzo[b]fluoren-11-one	-	-	1.21±0.03	-	-	-	0.53±0.01	-	-	-	-	-	-	-	-	-	-	-	-
316	4-methyl-Benzamide	-	-	-	-	3.80±0.12	1.36±0.26	-	-	-	-	-	-	-	-	-	-	-	-	-
317	Durophenol	-	-	-	-	31.40±3.68	16.98±1.32	-	-	-	-	-	-	1.42±0.05	-	-	1.54±0.01	-	-	-

Table S2. (continued).

No.	Compound	Concentration (µg/g)																		
		**WZM**	**SMQ**	**XQG**	**NFM**	**GC**	**XJ**	**XN**	**ZM**	**CX**	**HY**	**DD**	**PS**	**LZ**	**BT**	**JP**	**XC**	**LF**	**YF**	**NHE**
318	(2-Nonyloxy-benzyl)-phenyl-amine	-	-	5.96±0.32	-	-	-	-	-	-	-	-	-	-	-	-	-	-	-	-
319	13H-Dibenzo[a,i]carbazole	-	-	-	-	-	-	-	-	-	-	-	-	-	-	2.15±0.32	-	1.17±0.32	0.68±0.02	-

320	2-[1-(2-phenylethyl)-2-benzimidazolylmethylthio]-Benzothiazole	-	-	-	-	-	-	-	-	-	-	-	-	-	-	1.17±0.02	-	-	-	-
321	5-ethyl-3-(3-methyl-5-phenylpyrazol-1-yl)-1,2,4-Triazol-4-amine	1.34±0.01	-	-	-	-	-	-	1.72±0.32	-	-	-	-	-	-	-	-	-	-	-
322	2-Nitrobenzenesulfonohydrazide	-	-	-	-	-	-	-	-	-	-	-	-	3.12±0.12	-	-	-	-	-	-
323	1,3-dimethyl-5,6-dimethoxy-2-(3,5-dimethoxyphenyl)-1H-Indole	-	-	-	-	-	-	-	-	-	-	-	-	-	-	-	-	0.61±0.01	1.23±0.003	-
324	N-[4-[1-(3-trifluoromethylbenzyl)-2-benzimidazolyl]-3-furazanyl]-Acetamide	-	-	-	-	-	-	-	-	-	3.55±0.32	-	-	-	-	-	-	-	-	-
325	2-Amino-4-hydroxy-6-phenethyl pteridine	-	-	-	-	-	-	-	-	-	-	-	-	-	-	-	0.73±0.03	-	-	-
326	2-ethyl-4,5-dimethyl-Phenol	-	-	-	-	-	-	-	-	-	-	-	-	-	-	-	4.06±0.68	-	-	-
327	2,4-dimethyl-Benzo[h]quinoline	-	-	-	-	-	-	-	-	-	-	-	-	-	-	-	-	10.65±1.32	12.31±2.31	-
328	2-(4-Methoxy-phenoxy)-6-methyl-4-phenyl-quinazoline	-	-	-	-	-	-	-	-	-	-	-	-	-	-	-	-	2.32±0.06	1.29±0.31	-
329	N-[4-(1-methylethyl)benzylidene]-4-(1-pyrrolidylsulfonyl)-Benzenamine	-	-	-	-	-	-	-	-	-	-	-	-	-	-	-	-	0.12±0.001	0.67±0.01	-
330	5-(3-aminopropyl)-5,10-dihydro-11H-Dibenzo[b,e][1,4]diazepin-11-one	-	-	-	-	-	-	-	-	-	-	-	-	-	-	-	-	1.21±0.23	0.31±0.01	-
	Others																			
331	Dimethyl ether	-	-	10.13±0.31	-	1.36±0.04	2.54±0.05	-	-	-	-	-	-	-	-	1.01±0.02	-	-	-	-

332	2-methoxy-4-methyl-1-(1-methylethyl)-Benzene	-	-	-	5.15±0.12	-	-	5.78±0.14	3.91±0.31	2.21±0.04	1.57±0.01	-	-	2.18±0.35	-	3.69±0.45	-	1.69±0.21	3.97±0.31	4.13±1.01
333	Tripropylene glycol monomethyl ether	-	-	-	-	-	-	-	-	-	-	-	-	-	-	-	-	1.65±0 .07	1.36±0.12	-
334	1-methyl-1H-Pyrrole	-	-	-	-	6.79±0.68	8.21±1.65	-	-	-	-	-	-	-	2.65±0.04	-	-	-	-	-
335	1-chloro-2-nitro-Propane	-	-	-	-	-	-	-	-	-	-	-	-	-	-	-	-	12.23±1.08	6.74±0.62	-

Table S2. (continued).

No.	Compound	Concentration (μg/g)																		
		WZM	SMQ	XQG	NFM	GC	XJ	XN	ZM	CX	HY	DD	PS	LZ	BT	JP	XC	LF	YF	NHE
336	2-Methyl-3-trans-propenylpyrazine	-	-	-	-	-	-	-	-	-	-	-	-	-	6.12±0.27	-	-	-	-	2.77±0.09
337	1-chloro-2-nitro-1H-Pyrrole	-	-	-	-	4.26±0.12	9.00±1.23	-	-	-	-	-	-	-	18.08±0.68	-	-	-	-	-
338	2,3-dihydro-4-methyl-Furan	-	-	-	-	-	-	-	-	-	-	-	-	-	-	13.88±2.01	-	-	-	-
339	1,1'-dithiobis-Piperidine	-	-	-	-	-	-	-	-	-	-	-	-	-	-	-	15.91±1.89	-	-	-
340	Dimethoxyamine	-	0.30±0.001	-	-	-	-	0.14±0.001	-	-	-	-	-	-	-	-	-	-	-	-
341	1-Anthracenamine	-	0.26±0.001	-	-	-	-	-	-	-	-	-	-	-	-	-	-	0.64	1.03	-
342	1-ethyl-1H-Pyrrole	-	-	-	-	-	0.14±0.01	-	-	-	-	-	-	-	-	-	-	-	-	-

343	N-Benzylformamide	-	-	-	-	-	-	-	1.75±0.21	-	-	-	-	-	-	-	-	-	-	-
344	5H-Naphtho[2,3-c]carbazole	-	-	3.92±0.24	-	-	0.99±0.01	-	-	-	-	-	-	-	-	-	-	-	-	-
345	1-methyl-1H-Pyrazole	-	-	-	-	-	-	-	-	-	-	-	-	-	-	1.47±0.01	-	-	-	-
346	1-(5-trifluoromethyl-2-pyridyl)-4-(1H-pyrrol-1-yl)-Piperidine	-	-	-	1.40±0.03	-	-	-	-	-	-	-	-	-	-	0.75±0.02	-	-	-	-
347	(2-Aziridinylethyl)amine	-	-	-	-	-	-	-	-	-	-	-	-	-	0.78±0.12	-	-	-	-	-
348	1-ethyl-1H-Pyrrole-2-carboxaldehyde	-	-	-	-	3.45±0.42	2.77±0.35	-	-	-	-	-	-	-	-	-	-	-	-	-
349	5-methyl-1H-Indole	-	-	-	-	-	0.79±0.01	-	-	-	-	-	-	-	-	-	-	-	-	-
350	2-[N-Aziridyl]methylpyrrole	-	-	-	-	0.69±0.03	1.49±0.24	-	-	-	-	-	-	-	-	-	-	-	-	-
351	2-(methylamino)-Ethanol	0.59±0.04	-	-	-	-	-	-	-	1.57±0.12	-	-	-	-	-	-	-	-	-	-
352	Betazole	0.94±0.03	-	-	-	-	-	-	-	-	-	-	-	-	-	-	-	-	-	-
353	9-Vinylcarbazole	-	-	-	-	-	-	2.55±0.34	-	-	-	-	-	-	-	-	-	-	-	-
354	2-Propanamine	-	-	-	-	-	-	-	-	0.51±0.01	-	-	-	-	-	-	-	-	-	-
355	1,2-Hydrazinedicarboxamide	-	-	-	-	-	-	-	-	1.39±0.12	-	-	-	-	-	-	-	-	-	-
356	1-(1,1-Dioxo-tetrahydro-1.lambda.(6)-thiophene-3-sulfonyl)-piperidine	-	-	-	-	-	-	-	-	-	-	-	-	-	-	-	-	5.13±0.17	4.21±0.12	-

357	2-methoxy-Ethanamine	-	-	-	2.79±0.18	-	-	-	-	-	-	-	-	-	-	-	-	-	-	-
358	5H-Naphtho[2,3-b]carbazole	-	-	-	2.86±0.23	-	-	-	-	-	-	-	-	-	-	-	-	-	-	-

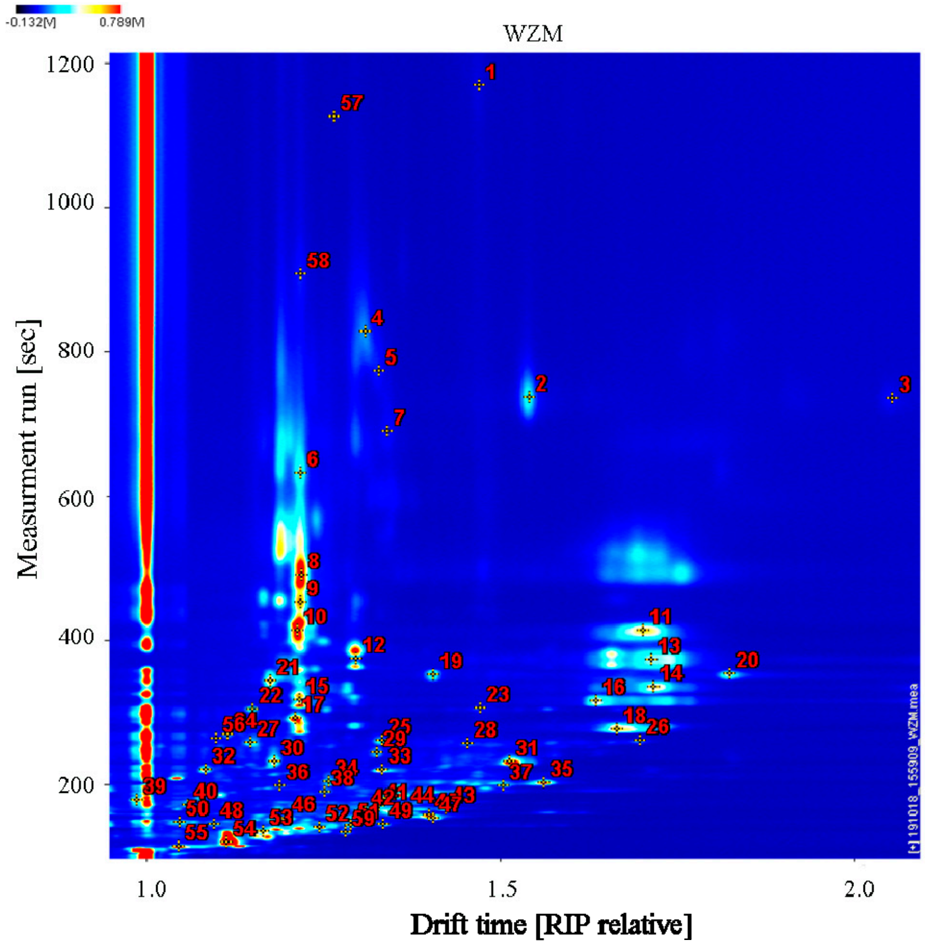

Figure S1. Ion migration spectra of WZM. The numbers are the identified volatile components.

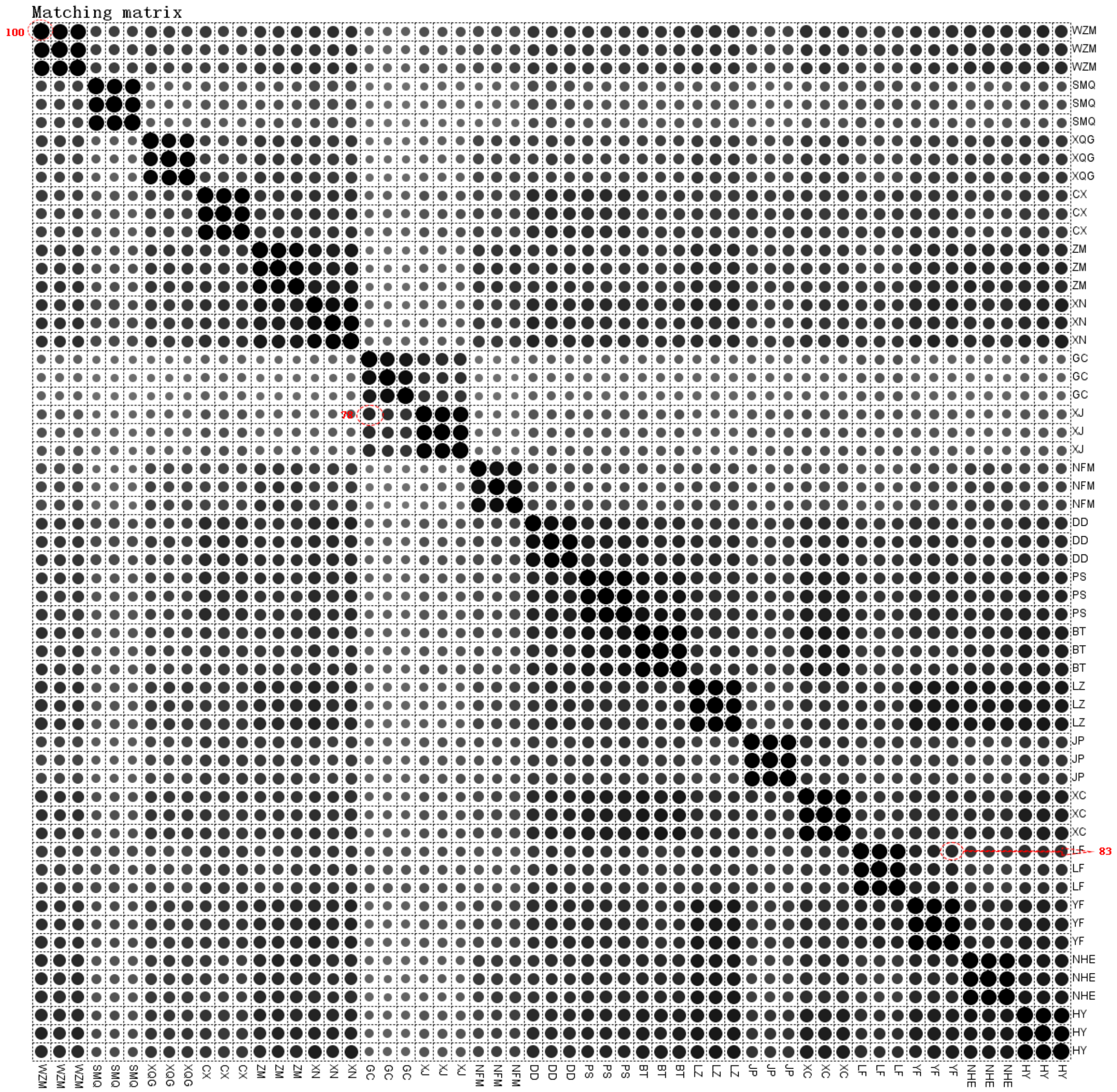

Figure S2. Similarity between samples of citrus-tea; the larger the dot, the higher the similarity.

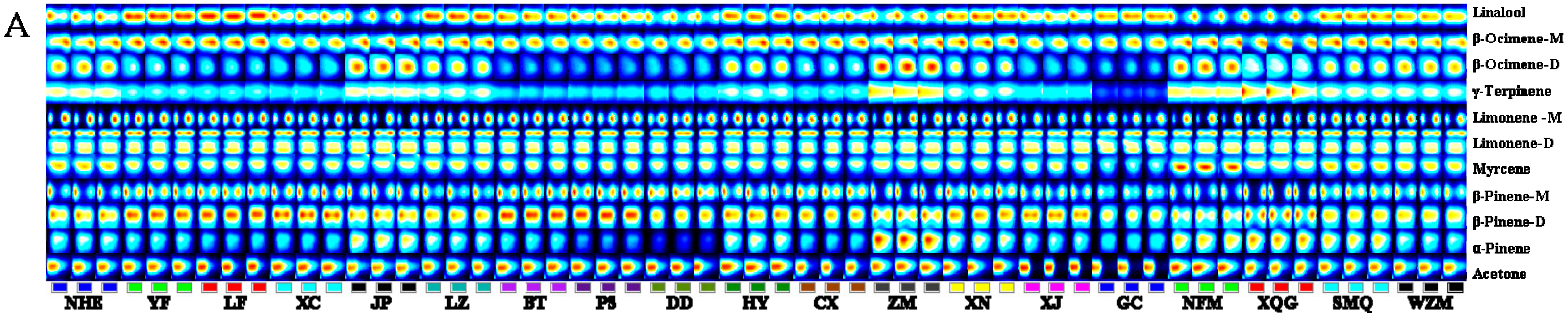

A
Linalool
β-Ocimene-M
β-Ocimene-D
γ-Terpinene
Limonene -M
Limonene-D
Myrcene
β-Pinene-M
β-Pinene-D
α-Pinene
Acetone
NHE
YF
LF
XC
JP
LZ
BT
PS
DD
HY
CX
ZM
XN
XJ
GC
NFM
XQG
SMQ
WZM

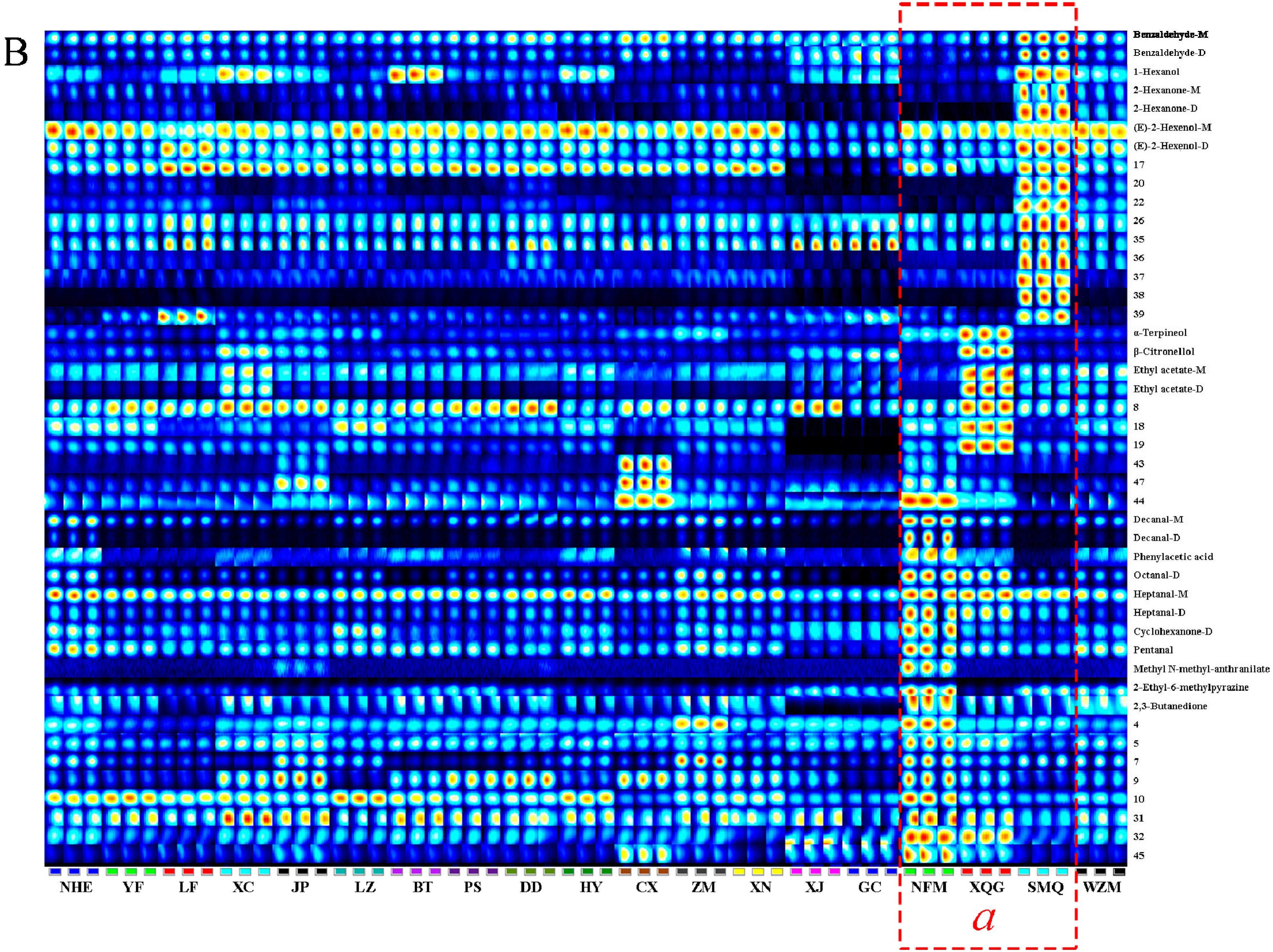
B
Benzaldehyde-M
Benzaldehyde-D
1-Hexanol
2-Hexanone-M
2-Hexanone-D
(E)-2-Hexenol-M
(E)-2-Hexenol-D
17
20
22
26
35
36
37
38
39
α-Terpineol
β-Citronellol
Ethyl acetate-M
Ethyl acetate-D
8
18
19
43
47
44
Decanal-M
Decanal-D
Phenylacetic acid
Octanal-D
Heptanal-M
Heptanal-D
Cyclohexanone-D
Pentanal
Methyl N-methyl-anthranilate
2-Ethyl-6-methylpyrazine
2,3-Butanedione
4
5
7
9
10
31
32
45
NHE
YF
LF
XC
JP
LZ
BT
PS
DD
HY
CX
ZM
XN
XJ
GC
NFM
XQG
SMQ
WZM
a

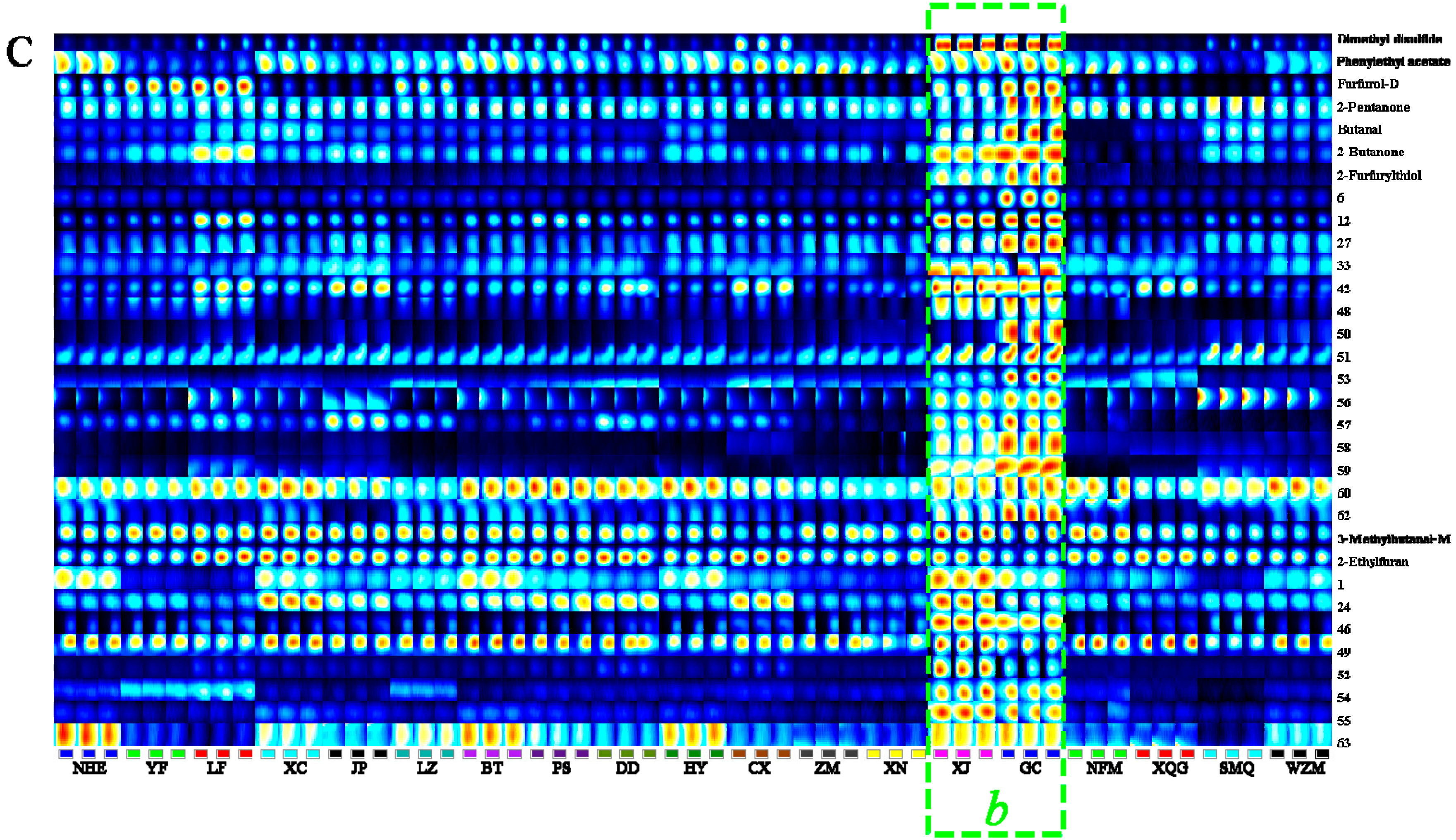

C
Dimethyl disulfide
Phenylethyl acetate
Furfurol-D
2-Pentanone
Butanal
2-Butanone
2-Furfurylthiol
6
12
27
33
42
48
50
51
53
56
57
58
59
60
62
3-Methylbutanal-M
2-Ethylfuran
1
24
46
49
52
54
55
63
NHE
YF
LF
XC
JP
LZ
BT
PS
DD
HY
CX
ZM
XN
XJ
GC
NFM
XQG
SMQ
WZM
b

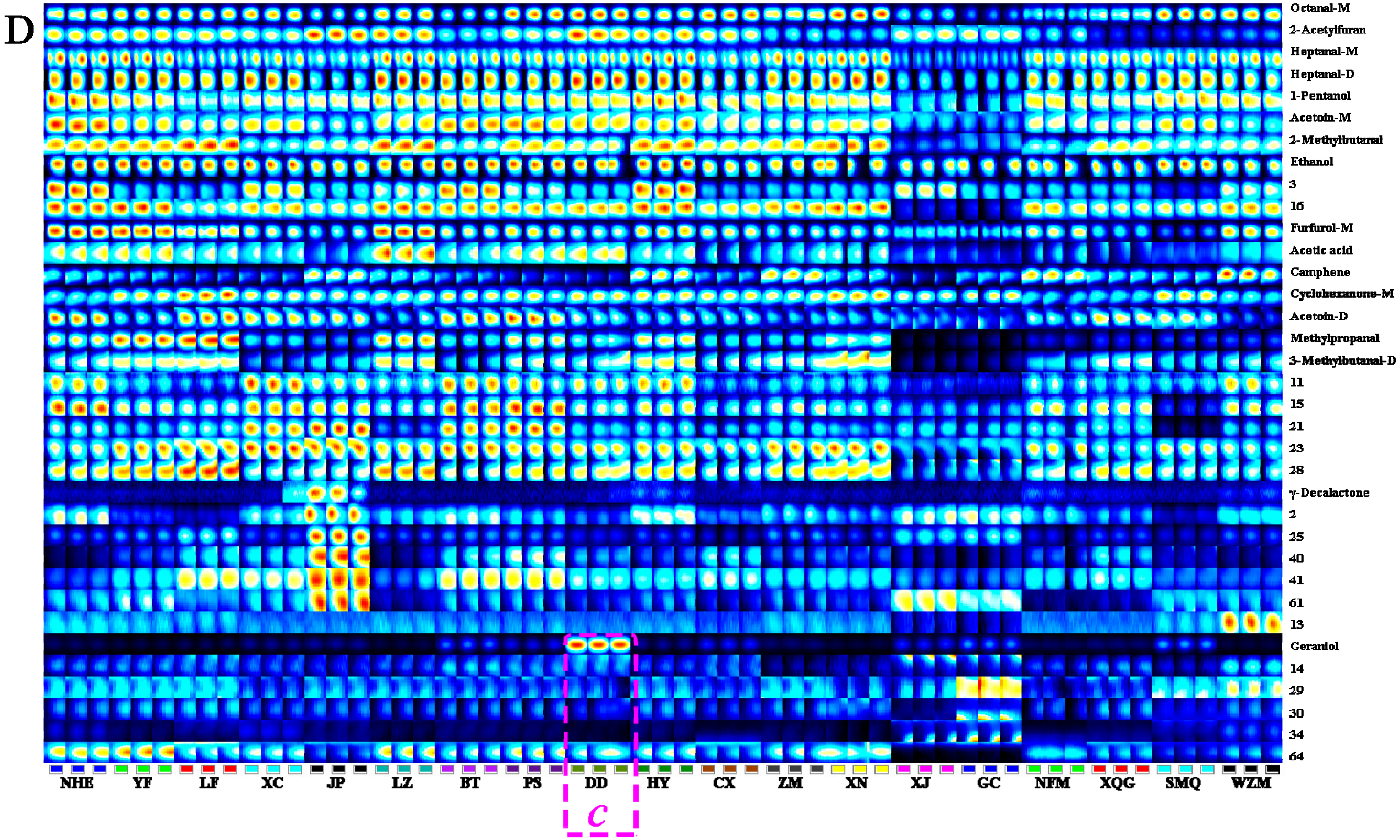

Figure S3. Four parts of the screenshots from the Gallery Plot fingerprints. Eleven kinds of identified peaks (**a**); 22 kinds of identified peaks (**b**); 9 kinds of identified peaks (**c**); 17 kinds of identified peaks (**d**).
